# Educational Interventions for Medical Students to Improve Pharmacological Knowledge and Prescribing Skills: A Scoping Review

**DOI:** 10.5334/pme.1006

**Published:** 2023-08-30

**Authors:** Weiwei Shi, Helen Qin, Brett Vaughan, Louisa Ng

**Affiliations:** 1 Melbourne Medical school, The University of Melbourne, Melbourne, Victoria, Australia; 2 Department of Medical Education, The University of Melbourne, Melbourne, Victoria, Australia; 3 The University of Melbourne, Melbourne Medical school, and Department of Medical Education, Australia; 4 Department of Rehabilitation Medicine, The Royal Melbourne Hospital, Melbourne, Victoria, Australia

## Abstract

**Introduction:**

Medication-related errors place a heavy financial burden on healthcare systems worldwide, and mistakes are most likely to occur at the stage of prescribing. Junior doctors are more likely to make prescribing errors, and medical graduates also lack confidence and preparedness towards prescribing. Thus, this review aimed to evaluate the existing educational approaches to improve pharmacological knowledge and prescribing skills among medical students.

**Methods:**

CENTRAL, CINAHL, ERIC, Ovid Embase, Ovid MEDLINE, Ovid PsycINFO, and Scopus were searched with keywords related to “pharmacological knowledge”, “prescribing skills”, “educational interventions” for articles published since 2016.

**Results:**

3595 records were identified, and 115 full-text articles were assessed for eligibility. Eighty full-text articles were eligible and included in this review. Thirty-seven studies focused on improving prescribing skills, whilst 43 targeted pharmacological knowledge. A broad range of interventions was implemented, including e-learning, case-based, interprofessional, and experiential learning. Pharmacological knowledge and prescribing skills were measured in various ways, and all studies reported one or more positive findings at Kirkpatrick level 1 or 2. No study reported outcomes at Kirkpatrick levels 3 and 4.

**Discussion:**

The World Health Organisation’s Good Guide to Prescribing was the foundation of the development of prescribing educational interventions. Emerging interventions such as experiential and interprofessional learning should be incorporated into the prescribing curriculum. Innovative approaches such as game-based learning can be considered for clinical pharmacology teaching. However, there was a lack of outcomes at Kirkpatrick levels 3 and 4. Robust methodology and reliable outcome measures are also needed in future studies.

## Introduction

Medication-related errors and unsafe prescribing practices have been recognised as one of the leading causes of preventable harm in healthcare systems globally. The World Health Organisation (WHO) estimated that the cost globally associated with medication-related errors is up to USD$42 billion annually [1]. Although medication-related errors can occur at any point from the prescription process through to administration, errors are most likely to take place during prescribing [2]. It has been estimated that prescribing errors occur in 7% of total medication orders and up to 50% of hospital admissions. Of these prescribing errors, those related to medication dosage and antimicrobial use are most reported [3].

In the past decade, two large studies found that doctors in their first two years of training were far more likely to make a prescribing error than senior doctors [4,5]. These studies identified that although the most frequent error-causing factor was the busy and complex working environment, at least 25% of the junior doctors perceived a lack of knowledge or experience as a significant factor. A more recent systematic review of final-year medical student prescribing competency concluded that medical graduates lacked prescribing competencies necessary for safe prescribing as well as self-confidence and self-perceived preparedness [6]. The 2019 Australian National Preparedness for Internship Survey and a large European cross-sectional study also reported similar findings [7].

In *Tomorrow’s Doctors* 2009, the United Kingdom General Medical Council states that medical graduates are expected to “prescribe drugs safely, effectively and economically” [8]. Therefore, medical schools play a pivotal role in preparing students for their role as prescribers. There have been significant changes in the delivery of clinical pharmacology and therapeutics education in medical schools over the last two decades, with a shift from didactic teaching to problem-based learning (PBL) as recommended by *Tomorrow’s Doctors* in 1993 [9]. As a result, pharmacology teaching is now commonly incorporated into the curriculum in the form of PBL rather than being delivered as an individual subject to reduce information overload [9]. With this shift, medical schools have attempted to determine the most effective educational approaches to improve pharmacological knowledge and prescribing skills among medical students. Three comprehensive reviews on educational interventions to improve prescribing skills have been published since 2009, all acknowledging the effectiveness of using the WHO Good Guide to Prescribing (GGP) as the cornerstone of prescribing curriculum design [10,11,12]. This six-step approach to prescribing has been utilised to develop other clinical pharmacology educational programs, such as the National Prescribing Service MedicineWise in Australia, a comprehensive online learning program [11,13]. One of the reviews also found emerging evidence of the benefits of promoting published therapeutic guidelines and optimising interprofessional communication [11]. The most recent review additionally noted the effectiveness of small-group teaching. However, the authors also highlighted the lack of innovation in prescribing education and longitudinal follow-up regarding the effectiveness of prescribing educational interventions [12].

Notably, most reviews to date have included participants in the early stages of their careers (i.e., qualified doctors but within two years post-graduation). Whilst medical students have been included in some of these reviews, none have specifically targeted this population. Most of the literature focuses solely on prescribing skills rather than pharmacological knowledge. There is no doubt that prescribing is a complex task requiring the integration of knowledge, skills and attitudes of clinical pharmacology and therapeutics [14]. However, pharmacology acts as the scientific basis of safe prescribing in clinical practice [15]. Therefore, it is crucial for medical students to improve their theoretical knowledge alongside the development of necessary skills and attitudes for safe prescribing within an interactive clinical context [16]. As the body of research in this field grows, a comprehensive analysis of contemporary educational approaches in clinical pharmacology and prescribing is needed to inform medical educators of new and innovative approaches. Therefore, this scoping review aims to provide a contemporary and comprehensive description of the range of prescribing and pharmacology educational approaches and outcomes for medical students to guide medical educators and provide future research directions.

## Methods

The scoping review was conducted in accordance with the Joanna Briggs Institute methodology for scoping reviews [17].

### Search Strategy

The search strategy aimed to locate both published and unpublished studies. An initial limited search of Ovid MEDLINE and ERIC was undertaken to identify articles on the topic. The initial search strategy was developed with the aid of two librarians from the University of Melbourne and the Royal Melbourne Hospital respectively. The text words contained in the titles and abstracts of relevant articles and the index terms used to describe the articles were used to develop a full search strategy for CENTRAL, CINAHL, ERIC, Ovid Embase, Ovid MEDLINE, Ovid PsycINFO, and Scopus (see Appendix 1). Keywords and Medical Subjective Headings (MeSH) terms used included pharmacology, pharmaceutical, prescribing, teaching, education, learning, and undergraduate and postgraduate medical students. The search strategy, including all identified keywords and index terms, was adapted for each included database. The reference list of all included sources of evidence was screened for additional studies. Grey literature was searched using OpenGrey (http://www.opengrey.eu).

### Inclusion and Exclusion Criteria

All primary studies published in English from January 2016 to July 2021, with medical students as participants, were included. The date range ensured that included studies would be contemporary, and the initial limited search had demonstrated that a broad range of studies would be included. Included studies targeted clinical pharmacological knowledge and/or prescribing skills with outcomes classifiable by Kirkpatrick evaluation levels (See Table 1). We included studies that targeted any component of the prescribing task, such as drug choice, medication safety, and medication communication skills [18]. Conference papers/abstracts and opinion letters/commentaries were excluded.

**Table 1:** Kirkpatrick levels of assessing educational outcomes.


**Level 1: Reaction**	**Level 1a:** Satisfaction reactions, commonly described as “liking of training”.

	**Level 1b:** Utility reactions, which are self-perceived or self-assessed and include usefulness of the intervention, “ability to perform the job” and confidence

**Level 2: Learning**	**Level 2a:** Changes in attitudes or perceptions **Level 2b:** Post-intervention knowledge **Level 2c:** Behaviour or skill demonstration

**Level 3: Transfer**	**Level 3:** Transfer of attitudes or perceptions, knowledge, and skills into workplace

**Level 4: Results**	**Level 4a:** Changes in organisational practice including changes within the organisation or delivery of care **Level 4b:** Benefits to patients including improvement in the health outcomes and well-being of the patients


*Note*: Adapted from a meta-analysis [102] of the relations among training criteria and a research article published by Yardley and Dornan [103] on Medical Education.

All titles and abstracts were independently screened by two review authors (WS and HQ) based on Preferred Reporting Items for Systematic Reviews and Meta-Analyses guidelines [19]. Full-text articles were subsequently retrieved and reviewed for final inclusion. Any uncertainty was resolved by discussion among the four authors (WS, LN, HQ, BV).

### Data Extraction

Study design, participants, intervention, outcome assessments, assessment time points and results were independently extracted by WS and HQ.

#### Reflexive Statement

The research question was derived from the author’s experience as medical educators and medical students, namely the challenge to learn and teach clinical pharmacology and prescribing skills. Further, our interest was in understanding how best to engage learners with this content. Therefore, the search strategy was developed in the light of the authors’ joint interests. The two authors who undertook the data extraction (WS, HQ) were medical students at the time, and the extraction was verified by the other authors (LN, BV). The inclusion of a non-medical practitioner who had not taught this content (BV) ensured a level of rigour in the data extraction oversight and subsequent analysis.

## Results

### Study characteristics

**Figure 1:**
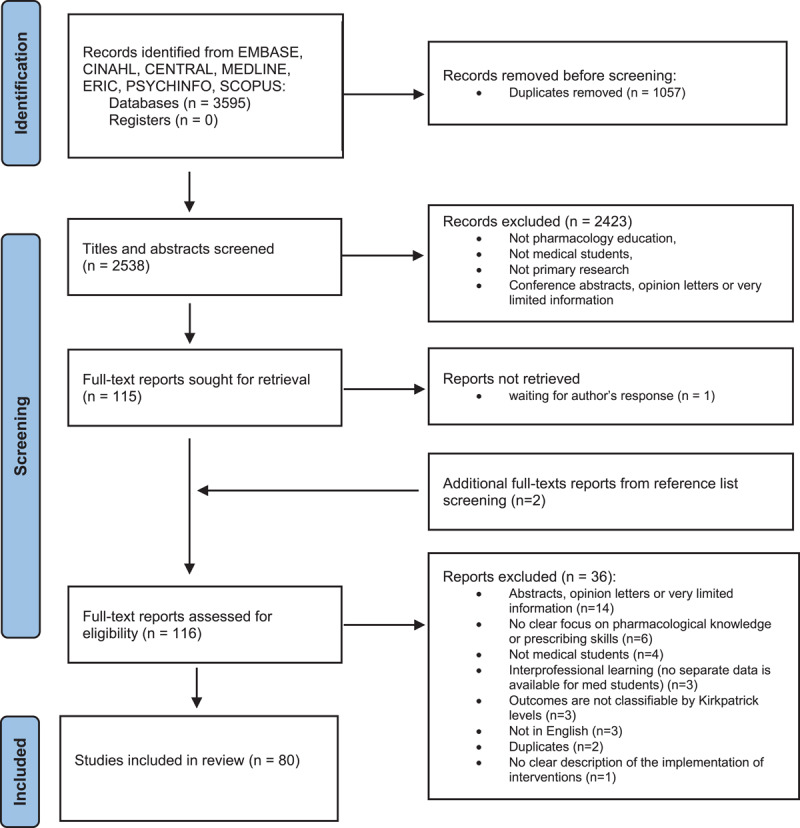
PRISMA flow chart.

In total, 3595 records were identified through the initial search. Following the screening, 2423 records were excluded. One hundred and sixteen (n = 116) full-text articles were assessed for eligibility (See Figure 1), and 80 studies met the inclusion criteria. For details of each study see Appendix 2. Almost half of the studies (34 studies, 43%) were conducted in India.

There was a mix of study designs, including eight randomised controlled trials [20,21,22,23,24,25,26,27], nine comparative studies [28,29,30,31,32,33,34,35,36], six prospective studies [37,38,39,40,41,42], 12 before and after studies [43,44,45,46,47,48,49,50,51,52,53,54], five mixed-method evaluation studies [55,56,57,58,59], five crossover studies [60,61,62,63,64], five quasi-experimental studies [65,66,67,68,69] and four cross-sectional studies [70,71,72,73]. The remaining 26 were post-intervention evaluation studies. There was a total of 11305 participants (not including the three studies that did not specify the number of participants), with each study including between six and 606 participants. Participants were spread across all year levels, with the highest number (n = 549) of participants in their second year of medical school. Forty-three studies aimed to improve clinical pharmacological knowledge, whilst 37 studies prioritised improving prescribing skills.

Whilst most studies only reported immediate outcomes, eight included delayed outcomes (ranging from three days to four years). Eleven studies utilised a combination of educational interventions.

### Assessment outcomes

Assessed outcomes were based on the first two levels of the Kirkpatrick training evaluation model. Sixty studies (75%) used objective assessments (Kirkpatrick level 2), including prescription writing and knowledge tests such as multiple-choice questions (MCQs), short answer questions (SAQs), and objective structured clinical examinations (OSCEs). Among these, 48 studies measured post-intervention knowledge (level 2b), 13 studies assessed prescribing skills (level 2c), and six studies evaluated students’ perceptions and attitudes towards the intervention (level 2a). Most studies (73 studies, 91%) included a form of subjective student evaluation (Kirkpatrick level 1) of the intervention using Likert scale questionnaires, feedback surveys or focus group interviews. 35 studies measured student satisfaction (level 1a), and 55 measured students’ evaluation of the usefulness and self-perceived confidence (level 1b). Four studies measured outcomes at level 2a. All studies reported positive findings for at least one assessed outcome; of these, 51 studies (64%) included an analysis for statistical significance.

### Types of intervention

A wide variety of educational interventions were implemented. E-learning was the most widely adopted strategy (16 studies, 20%), followed by case-based learning (10 studies, 12.5%) and simulation and role play (6 studies, 8%). Eight interventions (10%) were on interprofessional learning and six studies (8%) adopted experiential (patient-based) learning. There were five studies on game-based learning (6%) and three (4%) on peer-led learning. Eleven studies (14%) used a combination of interventions, including didactic learning, case-based learning, prescription writing exercises, and e-learning. Other studies implemented creative interventions such as video shooting and poem writing.

#### E-learning

Electronic learning, also known as ‘e-learning’ or “online learning”, was the most commonly evaluated intervention, with 16 studies investigating its effects [20,21,28,29,30,31,37,38,43,44,45,55,63,65,70,74]. E-learning is defined as the utilisation of the Internet and computing for educational purposes [75]. Seven studies [28,29,30,31,43,65,70] utilised computer-assisted learning in experimental pharmacology, and of these, two [31,70] used this approach to replace traditional animal experimental teaching and achieved positive outcomes in both post-intervention knowledge tests and satisfaction among students. Overall, students preferred computer-based animal simulation over animal experiments as they found the simulation more engaging and provided a clearer understanding of the topic. In addition, three studies implemented computer-assisted learning to teach prescribing, which combined theoretical content delivery with interactive case-based learning [20,21,38]. All three studies reported positive outcomes immediately post-intervention. Moreover, two of the three studies measured delayed outcomes and found higher knowledge retention among students from the intervention group compared to the control group at 30 days and 6 months, respectively [20,28]. Sengupta et al. [28] conducted a one-hour e-learning session and reported that the intervention group retained knowledge significantly better 30 days after the intervention than the control group who received one-hour didactic teaching. Similarly, Sikkens et al. [20] allowed students to complete the online learning over six weeks and found that the intervention group performed better in both knowledge tests and objective structured clinical examination (OSCE) prescription writing after six months than the control group who received no e-learning. Additionally, Tripathi et al. [37] compared the effectiveness and students’ perception of blended learning (didactic learning followed by e-learning) with e-learning alone. These authors reported that students found blended learning more enjoyable, whereas e-learning was more effective in knowledge gain.

#### Interprofessional learning

Eight studies examined the effectiveness of interprofessional learning on prescribing skills and found positive Kirkpatrick level 1 outcomes for all studies [47,48,49,56,57,76,77,78]. Of these, three studies [47,48,76] were pharmacist-led, and the remaining focused on interprofessional learning among pharmacy and medical students. Four studies described prescribing workshops where medical students participated in prescribing exercises with the support of pharmacists or pharmacy students [48,49,76,78]. Newby et al. implemented an eight-week pharmacist-led prescribing program in hospitals where students received feedback from pharmacists on their written prescriptions and participated in case-based tutorials [48]. Although there was no statistically significant improvement in the appropriateness of prescribing, students reported significant improvement in self-confidence and awareness of the good prescribing practice (p < 0.05).

#### Case-based learning

Kirkpatrick level 1 outcomes were positive for most studies evaluating case-based learning [33,34,50,60,66,79,80,81]. Brinkman et al. examined the effectiveness of integrated, case-based learning where pharmacology teaching was integrated with other disciplines, such as pathophysiology and microbiology [34]. It was reported that students from the integrated, case-based learning performed significantly better in both knowledge and prescribing tests.

#### Peer-led learning

Two RCTs compared the effectiveness of didactic tutorials with student-led objective tutorials — a form of self-directed learning where students engage in group learning with resources and guidance provided by educators [22,23]. More specifically, students in groups designed their own MCQs with self-determined learning objectives on the selected pharmacology topics and then presented the questions to the whole class [23]. Both RCTs reported that medical students perceived peer-led learning in small groups as more stimulating than didactic teaching, and their pharmacological knowledge improved post-intervention. Shenoy et al. compared the effectiveness of a student-led objective tutorial with a crossword puzzle in clinical pharmacology teaching [35]. It was reported that students from the student-led objective tutorial group scored significantly higher on the knowledge test, and they found student-led objective tutorial provided more in-depth learning than the crossword puzzle.

#### Experiential learning

Five studies incorporated a component of patient interaction or hospital-based learning into the teaching of prescribing skills [36,40,61,82,83]. Two studies were conducted in a hospital setting where medical students were directly involved in the delivery of patient care [40,82]. Kinston et al. provided students with opportunities to write prescriptions for inpatients under supervision. During the intervention, they conducted an audit of prescriptions and identified the common mistakes made during the process of prescribing. The study only reported Kirkpatrick level 1 outcomes which showed increased prescribing confidence. In the other study, students were involved in screening potential adverse drug reactions among inpatients, patient interviews and reporting of adverse drug reactions on the ward [40]. This intervention was reported to improve knowledge of objective assessments and raised awareness of the importance of adverse drug reaction recognition in clinical practice. Thenrajan et al. conducted a comparative study where the intervention group took patient histories whilst the control group was given written scenarios on the same clinical conditions [36]. The intervention group performed significantly better in their prescribing test. As an alternative to clinical-placement-based experiential learning at a time when clinical placements were paused during the COVID-19 pandemic, Jose et al. developed a creative project where students practiced medication history-taking with their families and friends instead. They reported that the intervention was useful in understanding the theoretical components of clinical pharmacology [84].

#### Simulation and role-play

Three studies aimed to improve pharmacological knowledge through simulations [31,39,85]. Arcoraci et al. conducted a comparative study involving 90 students and found that high-fidelity simulation was associated with effective learning and knowledge retention [32]. Nicolaou et al. reported that the interactive computerised simulation tool was more helpful in improving pharmacological knowledge than case-based learning [39]. Three other studies implemented peer role-play to enhance students’ medication communication skills [46,86,87]. Overall, medical students were satisfied with role-play as a learning tool and increased self-perceived confidence or competence using this educational strategy.

#### Game-based learning

Five studies were conducted to evaluate the effects of game-based learning on clinical pharmacology teaching [24,26,51,58,88]. Of these, four studies focused on pharmacological knowledge and reported positive Kirkpatrick level 1 and 2 outcomes. Interestingly, although one study found no difference in knowledge test results immediately post-intervention between groups (both groups improved, and the control group received didactic teaching), students who received game-based learning retained knowledge significantly better three days after the intervention [58].

#### Multicomponent educational interventions

Eleven studies used multicomponent interventions [42,52,53,67,71,89,90,91,92,93,94], and of these, seven focused on prescribing skills and four on clinical pharmacology knowledge. Among all seven studies on prescribing skills, the most common components included prescription writing exercises (4 studies) [42,53,71,89], case-based learning (3 studies) [90,91,94], and group discussions (3 studies) [42,53,71]. Three studies reported positive Kirkpatrick level 2c outcomes – statistically significant improvement in prescription writing and medication communication skills [53,71,94] and another reported retention of prescribing skills four years after the intervention (42). As for the four studies which examined the effectiveness of multicomponent educational interventions in the clinical pharmacology teaching [52,67,92,93], all four included a component of didactic teaching and reported at least one positive finding on Kirkpatrick level 1. Three of the studies also incorporated group learning in the format of group assignments or group discussions [52,92,93].

#### Other types of educational interventions

Three studies [95,96,97] evaluated creative interventions — the effects of video shooting, poem writing and creation of medication autobiographies in pharmacology teaching, respectively. All reported positive findings on Kirkpatrick level 1, with most students finding the interventions enjoyable. However, no outcomes at higher Kirkpatrick levels (including level 2) were assessed [95,96,97]. Moreover, two other studies introduced mind mapping as a learning tool which students found useful in facilitating their learning of clinical pharmacology [68,73].

## Discussion

This contemporary scoping review of pharmacology and prescribing education for medical students included 80 studies published in the past five years with a wide range of educational interventions. Kirkpatrick level 1 and 2 outcomes were widely found to be positive across interventions, but no studies evaluated Kirkpatrick level 3 or 4 outcomes.

Overall, the outcomes reported in the current review were similar to previous reviews on educational interventions for improving prescribing skills [10,11,12]. Over the last two decades, the WHO GGP was widely reported as one of the cornerstones of prescribing education, and this is consistent with the findings of this review [10,11,12]. Thirty-five studies included in the current review aimed to improve prescribing skills, and six of these explicitly reported designing their educational interventions based on the WHO GGP six-step approach [20,36,53,64,80,89]. All six studies reported positive Kirkpatrick level 1 outcomes and four of these also reported positive Kirkpatrick level 2 findings [20,36,53,64]. Moreover, problem-based learning underpinned by the WHO GGP appears to be an effective educational strategy [98]. Although other studies did not explicitly mention the WHO GGP, it was evident they incorporated some, if not all, of the WHO GGP principles, as clinical case scenarios formed the foundation of their interventions.

Experiential learning has not been emphasised in previous reviews [10,11,12] and appears to be an emerging approach The lack of emphasis on experiential learning is likely due to medical students being legally prevented from prescribing. However, the studies on experiential learning reported benefits with respect to improving prescribing skills and self-confidence, which highlights the importance of creating opportunities where possible for students to practice prescribing on the wards under supervision [82]. Auditing appears to be another effective intervention as by having medical students detect adverse drug reactions in physicians’ prescriptions, there were effective dual outcomes in terms of cost-effectiveness and improvement in prescribing skills [40]. As advocated by Linton and Murdoch-Eaton, prescribing training should occur in the context of a clinical environment to provide students with the most authentic experience possible [99]. Moreover, according to the 2019 Australian National Preparedness for Internship Survey, final-year students ranked “exposure to prescribing in clinical situations” as the third most effective factor in increasing preparedness [7]. Experiential learning should therefore be incorporated into existing prescribing curricula. For example, educators might consider that the increasing adoption of electronic medical record systems may provide a system of safe student prescribing through built-in safety checks by supervising doctors and pharmacists.

Prescribing education presents an opportunity to utilise interprofessional learning, given it reflects the reality of prescribing practice. Interprofessional learning has been implemented in the format of either pharmacist-led learning or interprofessional workshops with pharmacy students. All seven studies included in this review also incorporated a component of case-based learning [47,48,49,56,57,76,77]. Similar to previous reviews, all seven studies found positive Kirkpatrick level 1 outcomes. However, in contrast to other interventions, outcomes at Kirkpatrick level 2 were less promising due to a lack of p-values or statistical significance. Therefore, the lack of strong statistical evidence at Kirkpatrick level 2 and above in interprofessional learning should be addressed in future studies.

In contrast to other reviews, the current review examined the effects of educational strategies on improving pharmacological knowledge. There was a huge diversity of tools implemented, including game-based learning, simulation, video production and poem writing. Among these, game-based learning appeared effective not only in improving pharmacology knowledge [24,26,58,88], but engaging students in active learning [24]. The understanding of pharmacological concepts was reinforced through group discussion and collaboration with peers [58], demonstrating that whilst pharmacology may have traditionally been considered a ‘dry’ subject, there are educational approaches which can nevertheless be stimulating. Game-based learning also generates a contextualised environment, which promotes ‘comprehension-based acquisition of pharmacology knowledge’ rather than ‘linear order of knowledge gain’ [26,58]. Therefore, game-based learning could be a useful educational approach for stimulating interest in ‘dry’ topics [24] and reinforcing students’ newly acquired knowledge.

Furthermore, it was noted that e-learning [20,21,28,29,30,31,37,38,43,44,45,55,63,65,70,74] was one of the most common educational interventions for both clinical pharmacology and prescribing education likely due to increasing accessibility and cost-effectiveness. Seven [28,29,30,31,43,65,70] out of 16 studies using e-learning utilised a computer-assisted animal simulation in pharmacology teaching and achieved positive outcomes on Kirkpatrick level 1 or 2. All these studies were conducted in India, reportedly due to growing ethical concerns about performing animal experiments [28]. Other common forms of e-learning include online interactive modules with clinical cases and multimedia such as videos and animations. One of the pitfalls of e-learning is recognised as the lack of active interaction among peers and teachers [100]. One way of tackling this barrier could be blended learning, where traditional classroom teaching is combined with e-learning. However, this review only found one study that showed students preferred blended learning to traditional learning and pure e-learning [37].

There was a large variety of outcome measurements used to assess knowledge and prescribing skills, many of which appeared to be locally developed. One study [50] implemented team-based learning aiming to improve students’ preparedness towards the Prescribing Safety Assessment (PSA), which is a standardised assessment developed by the British Pharmacological Society to assess the prescribing skills among UK medical graduates [50]. The PSA was first delivered in 2016 and subsequently adopted in Australia and New Zealand, where the test has been regionalised and endorsed as an appropriate measure of prescribing competency [101]. Previous reviews have highlighted the lack of reliability and consistency in outcome assessments [10,11]. Therefore, as a comprehensive existing validated outcome assessment, the PSA could be considered as an outcome measure for future studies. However, it may be subject to resourcing limits, takes two hours to complete and incurs a cost.

The limitations of the current review included: methodological weaknesses common to most studies, such as lack of randomisation, blinding, and control groups. The heterogeneity of the study designs and outcome measures made comparisons challenging, and whilst satisfaction and utility reactions are important for student engagement, these correlate poorly to knowledge improvement or prescribing behaviour change [102]. Furthermore, all included studies reported at least one positive outcome, raising the possibility of publication bias.

This scoping review highlighted significant gaps in the current literature which should be considered for future research: (1) using robust methodology with validated outcome measures, statistical analysis for significant differences and inclusion of effect sizes; (2) comparison of different types of interventions; (3) outcome assessments at Kirkpatrick levels 3 and 4; (4) delayed outcome measures to determine retention; and (5) studies specifically targeting interprofessional learning and experiential learning.

In conclusion, all studies in this review found positive outcomes across a broad range of interventions, with e-learning being the most common. The review has also highlighted interventions which are innovative and/or emerging in pharmacology education for medical students, such as experiential, interprofessional and game-based learning.

## Additional Files

The additional files for this article can be found as follows:

10.5334/pme.1006.s1 Appendix 1. Search strategies.

10.5334/pme.1006.s2 Appendix 2. Summary of pharmacology and prescribing educational interventions for medical students.
