# Supplementary material for: Educational Interventions for Medical Students to Improve Pharmacological Knowledge and Prescribing Skills: A Scoping Review
Source: Perspect Med Educ. 2023 Aug 30;12(1):348–60. doi: 10.5334/pme.1006 (PMC10473179; doi:10.5334/pme.1006)
Supplement: Appendix 1. — Search strategies. [file pme-12-1-1006-s1.pdf]

Appendix 1: Search strategies[1]

Ovid Medline <1946 to July 23, 2021>

1	clinical clerkship/ or exp education, medical, graduate/ or exp education, medical, undergraduate/ or exp Schools, Medical/ or exp Students, Medical/	139442
2	((((post-graduate or postgraduate) adj4 medical) or (under-graduate or undergraduate)) adj4 medical).mp. [mp=title, abstract, original title, name of substance word, subject heading word, floating sub-heading word, keyword heading word, organism supplementary concept word, protocol supplementary concept word, rare disease supplementary concept word, unique identifier, synonyms]	33856
3	((medic* adj4 student*) or (medic* adj4 school*)).mp. [mp=title, abstract, original title, name of substance word, subject heading word, floating sub-heading word, keyword heading word, organism supplementary concept word, protocol supplementary concept word, rare disease supplementary concept word, unique identifier, synonyms]	112763
4	exp *pharmacology/ or exp pharmacology, clinical/ or exp Drug Prescriptions/ or exp Medication Errors/ or exp inappropriate prescribing/ or exp Potentially Inappropriate Medication List/ or exp *prescriptions/ or exp drug utilization/ or exp Education, Pharmacy/	182009
5	exp teaching/ or exp teaching materials/ or exp education/ or exp learning/	1294632
6	((pharmacol* or pharmaceutical* or medication* or drug or drugs or prescri*) adj4 (educat* or teach* or train* or utili?ation or learning)).mp. [mp=title, abstract, original title, name of substance word, subject heading word, floating sub-heading word, keyword heading word, organism supplementary concept word, protocol supplementary concept word, rare disease supplementary concept word, unique identifier, synonyms]	46256
7	(drug information or therapeutic information or pharmaceutical information).mp. [mp=title, abstract, original title, name of substance word, subject heading word, floating sub-heading word, keyword heading word, organism supplementary concept word, protocol supplementary concept word, rare disease supplementary concept word, unique identifier, synonyms]	6789
8	1 or 2 or 3	186884
9	4 and 5	16540
10	6 or 7 or 9	65017
11	8 and 10	2755
12	limit 11 to english language	2441
13	limit 12 to yr="2011 -Current"	1160

[1] All the search strategies were initially conducted to screen papers from 2011, however, with further discussion among all authors, the date range was further limited to Jan 2016 to July 2021 to ensure that included studies would be contemporary and the initial limited search had demonstrated that a broad range of studies would be included. All papers from 2011 to 2015 were manually excluded from Covidence prior to screening process.

Embase Classic & Embase <1947 to 2021 Week 29>

1	*medical education/ or exp medical student/ or exp clinical education/ or exp medical school/	226732
2	((medic* adj4 student*) or (medic* adj4 school*)).mp. [mp=title, abstract, heading word, drug trade name, original title, device manufacturer, drug manufacturer, device trade name, keyword, floating subheading word, candidate term word]	181418
3	((((post-graduate or postgraduate) adj4 medical) or (under-graduate or undergraduate)) adj4 medical).mp.	16242
4	exp education/ or exp learning/ or exp teaching/	2009730
5	exp *pharmacology/ or exp clinical pharmacology/ or exp *prescription/ or exp medication error/ or exp inappropriate prescribing/ or exp drug utilization/	1449801
6	4 and 5	41779
7	((pharmacol* or pharmaceutical* or medication* or drug or drugs or prescri*) adj4 (educat* or teach* or train* or utili?ation or learning)).mp. [mp=title, abstract, heading word, drug trade name, original title, device manufacturer, drug manufacturer, device trade name, keyword, floating subheading word, candidate term word]	57672
8	(drug information or therapeutic information or pharmaceutical information).mp.	27631
9	1 or 2 or 3	275308
10	6 or 7 or 8	119965
11	9 and 10	5081
12	limit 11 to (english language and yr="2011 -Current")	2705

APA PsycInfo <1806 to July Week 3 2021>

1	exp *medical education/ or exp medical students/	29297
2	((((post-graduate or postgraduate) adj4 medical) or (under-graduate or undergraduate)) adj4 medical).mp. [mp=title, abstract, heading word, table of contents, key concepts, original title, tests & measures, mesh]	2305
3	((medic* adj4 student*) or (medic* adj4 school*) or academic medic*).mp. [mp=title, abstract, heading word, table of contents, key concepts, original title, tests & measures, mesh]	30431
4	exp *pharmacology/ or exp *drug therapy/ or exp prescription drugs/ or exp "prescribing (drugs)"/	140068
5	exp teaching/ or exp education/ or exp learning/	712488
6	4 and 5	3358
7	((pharmacol* or pharmaceutical* or medication* or drug or drugs or prescri*) adj4 (educat* or teach* or train* or utili?ation or learning)).mp. [mp=title, abstract, heading word, table of contents, key concepts, original title, tests & measures, mesh]	10474
8	(drug information or therapeutic information or pharmaceutical information).mp. [mp=title, abstract, heading word, table of contents, key concepts, original title, tests & measures, mesh]	456
9	1 or 2 or 3	42276
10	6 or 7 or 8	13645
11	9 and 10	788
12	limit 11 to yr="2011 -Current"	348
13	limit 12 to english language	337

CINAHL <30/7/2021>

1	(MM "Education, Medical") OR (MH "Students, Medical") OR (MH "Schools, Medical") OR (MH "Faculty, Medical")	39,791
2	(medic* N3 school*) OR (medic* N3 student*)	38,108
3	(post-graduate OR postgraduate) N3 medical) OR ((under-graduate or undergraduate) N3 medical)	3,417
4	(MM "Pharmacy and Pharmacology+") OR (MH "Drugs, Prescription+") OR (MH "Medication Errors+") OR (MH "Inappropriate Prescribing")	47,743
5	(MH "Education+") OR (MH "Curriculum+") OR (MH "Education, Clinical+") OR (MH "Learning Methods+") OR (MH "Teaching+")	966,344
6	MH "Education, Pharmacy"	1,904
7	((pharmacol* OR medication* OR drug OR drugs OR prescri") N3 (educat" OR teach* OR train* OR utili?ation OR learning)	21,191
8	(drug information OR therapeutic information OR pharmaceutical information)	8,968
9	S1 OR S2 OR S3	54,597
10	S4 AND S5	6,899
11	S6 OR S7 OR S8 OR S10	36,860
12	S9 AND S11	903
13	S12	576

ERIC <30/7/2021>

1	(medic* N3 student*) OR (medic* N3 school*) OR academic medic*)	11,930
2	DE "medical education" OR DE "Medical Students" OR DE "Medical School Faculty" OR DE "Graduate Medical Education" OR DE "Medical Schools"	13,739
3	((post-graduate OR postgraduate) N3 medical) OR ((under-graduate or undergraduate) N3 medical)	674
4	DE "Curriculum" OR DE "Student Teaching" OR DE "Teaching (Occupation)" OR DE "Learning" OR DE "Learning Activities"	75,208
5	DE "Pharmacology" OR DE "Drug Therapy" OR DE "Drug Use"	7,771
6	DE "Pharmaceutical Education* OR DE "Drug Education*	4,054
7	drug information OR therapeutic information OR pharmaceutical information	1,086
8	((pharmacol* OR medication* OR drug or drugs OR prescri*) N3 (educat* OR teach* OR train* OR utili?ation or learning)	7,266
9	S1 OR S2 OR S3	16,467
10	S4 AND S5	125
11	S6 OR S7 OR S8 OR S10	9,220
12	S9 AND S11	389
13	S12	90

Scopus <31/07/2023>

1	TITLE-ABS-KEY ((post-graduate W/3 medical) OR (postgraduate W|3 medical))	7,001
2	TITLE-ABS-KEY ((under-graduate WIS medical) OR (undergraduate W3 medical))	28,751
3	TITLE-ABS-KEY ((medic* WI student*) OR (medic* w3 school*))	178,341
4	TITLE-ABS-KEY ((drug AND information) OR (therapeutic AND information) OR (pharmaceutical AND information))	389,442
5	TITLE-ABS-KEY ((pharmacol* OR pharmaceutical* OR medication* OR drug OR drugs OR prescri*) W/3 (educat* OR teach* OR train* OR utili?ation OR learning))	63,164
6	(TITLE-ABS-KEY ((post-graduate W/3 medical) OR (postgraduate W/3 medical))) OR (TITLE-ABS-KEY ((under-graduate W/3 medical) OR (undergraduate W/ medical))) OR (TITLE-ABS-KEY ((medic* W/3 student*) OR (medic* W/3 school*)))	190,854
7	(TITLE-ABS-KEY ((drug AND information) OR (therapeutic AND information) OR (pharmaceutical AND information))) OR (TITLE-ABS-KEY ((pharmacol* OR pharmaceutical* OR medication* OR drug OR drugs OR prescri*) W/3 (educat* OR teach* OR train* OR utili?ation OR learning)))	442,989
8	((TITLE-ABS-KEY ((post-graduate W/3 medical) OR (postgraduate W/3 medical))) OR (TITLE-ABS-KEY ((under-graduate W/3 medical) OR (undergraduate W/3 medical))) OR (TITLE-ABS-KEY ((medic* W/3 student*) OR (medic* W/3 school*)))) AND ((TITLE-ABS-KEY ((drug AND information) OR (therapeutic AND information) OR (pharmaceutical AND information))) OR (TITLE-ABS-KEY ((pharmacol* OR pharmaceutical* OR medication* OR drug OR drugs OR prescri* w/ (educat* OR teach* OR train* OR utili?ation OR learning))))	3,291
9	((TITLE-ABS-KEY ((post-graduate W/3 medical) OR (postgraduate W/3 medical))) OR (TITLE-ABS-KEY ((under-graduate W/3 medical) OR (undergraduate W/3 medical))) OR (TITLE-ABS-KEY ((medic* W/3 student*) OR (medie* W/3 school*)))) AND ((TITLE-ABS-KEY ((drug AND information) OR (therapeutic	1,588

	AND information) OR (pharmaceutical AND information))) .OR (TITLE-ABS-KEY ((pharmacol* OR pharmaceutical* OR medication* OR drug OR drugs OR prescri*) W|3 (educat* OR teach* OR train* OR utili?ation OR learning)))) AND (LIMIT-TO (PUBYEAR, 2021) OR LIMIT-TO (PUBYEAR, 2020) OR LIMIT-TO (PUBYEAR, 2019) OR LIMIT-TO (PUBYEAR, 2018) OR LIMIT. TO (PUBYEAR , 2017) OR LIMIT-TO (PUBYEAR, 2016) OR LIMIT-TO (PUBYEAR, 2015) OR LIMIT-TO (PUBYEAR, 2014) OR LIMIT-TO (PUBYEAR, 2013) OR LIMIT-TO (PUBYEAR, 2012) OR LIMIT-TO (PUBYEAR, 2011))	
10	((TITLE-ABS-KEY ((post-graduate W/3 medical) OR (postgraduate W/3 medical))) OR (TITLE-ABS-KEY ((under-graduate W/3 medical) OR (undergraduate W/3 medical))) OR (TITLE-ABS-KEY ((medic* W/3 student*) OR (medic* W/3 school*)))) AND ((TITLE-ABS-KEY ((drug AND information) OR (therapeutic AND information) OR (pharmaceutical AND information))) .OR (TITLE-ABS-KEY ((pharmacol* OR pharmaceutical* OR medication* OR drug OR drugs OR prescri*) W|3 (educat* OR teach* OR train* OR utili?ation OR learning)))) AND (LIMIT-TO (PUBYEAR, 2021) OR LIMIT-TO (PUBYEAR, 2020) OR LIMIT-TO (PUBYEAR, 2019) OR LIMIT-TO (PUBYEAR, 2018) OR LIMIT. TO (PUBYEAR , 2017) OR LIMIT-TO (PUBYEAR, 2016) OR LIMIT-TO (PUBYEAR, 2015) OR LIMIT-TO (PUBYEAR, 2014) OR LIMIT-TO (PUBYEAR, 2013) OR LIMIT-TO (PUBYEAR, 2012) OR LIMIT-TO (PUBYEAR, 2011)) AND (LIMIT-TO (LANGUAGE , "English"))	1,467
