# Supplementary material for: Educational Interventions for Medical Students to Improve Pharmacological Knowledge and Prescribing Skills: A Scoping Review
Source: Perspect Med Educ. 2023 Aug 30;12(1):348–60. doi: 10.5334/pme.1006 (PMC10473179; doi:10.5334/pme.1006)
Supplement: Appendix 2. — Summary of pharmacology and prescribing educational interventions for medical students. [file pme-12-1-1006-s2.pdf]

Appendix 2: Summary of pharmacology and prescribing educational interventions for medical students

Study	Country	Study Design	Participants (n=)	Intervention	Focus Area	Outcome measures	Assessment timepoints	Kirkpatrick Level	Results
**E-learning**									
Amirtha et al. (2017) (43)	India	Before and after study	200 second-year medical students	Six 1-hour long animal stimulatory experiments with CAL[1]	Clinical pharmacology	**Objective:**<br>Knowledge test (further details not specified)<br><br>20-station OPSE[2] consisting of written quizzes, clinical case charts and CAL modules<br><br>CAL and general pharmacology viva-voce<br><br>**Subjective:** NA	Before and immediately after the intervention<br><br>Immediately after the intervention<br><br>Immediate after the intervention	Level 2b<br><br>Level 2b<br><br>Level 2b	Statistically significant improvement in written scores (p<0.01) following the intervention. In addition, after the intervention, more students scored >50% on the viva-voce examinations compared to the written examinations.
Sengupta et al. (2017) (28)	India	Comparative study	115 second-year undergraduate medical students<br><br>C = 55<br>I = 66	**C**: 1 hour of conventional didactic teaching on the effects of different medications on mean arterial blood pressure<br><br>**I:** 1 hour of computer-assisted learning on the same topic	Clinical pharmacology	**Objective**:<br>Knowledge tests (15 MCQ[3]s)<br><br>**Subjective**:<br>Likert scale student evaluation on the learning experience	Immediately after intervention and 30 days after intervention<br><br>Immediately after intervention	Level 2b<br><br>Level 1a	No statistically significant differences between two groups immediately after intervention (p=0.126). Intervention group retained the knowledge (30 days after) better than the control group (p=0.001).<br>No statistically significant differences (p=0.6033)
Tripathi et al. (2017) (37)	India	Prospective single group study	180 second-year undergraduate medical students	1. Pre-supplemental model: internet resources (typenot specified) were provided to students before the didactic lecture (antithyroid drugs)<br><br>2. Post-supplemental model: the didactic lecture was given before internet resources were made available (psychopharmacology)<br><br>3. replacement model: internet resources and didactic lecture slides were provided to students (no didactic lecture was delivered) on the topic of anthelminthic drugs<br><br>*One month gap between each model	Clinical Pharmacology	**Objective**:<br>Pre-and post-intervention knowledge tests for each model (40 MCQs)<br><br>**Subjective**:<br>Student evaluation of the intervention	Before and on completion of the intervention (≤ 8 days)<br><br>On completion of the intervention	Level 2b<br><br>Level 1a, 1b	Students performed significantly better in the post-intervention tests across all three models (p<0.01). Students from model 3 scored the highest comparatively (no p-value provided).<br>Overall, more than 50% of the students agreed that they were satisfied with e-learning, and it motivated them to self-learning. Around 59% of them opined that the knowledge acquired by e-learning would be helpful in clinical practice and 73% believed that e-learning should only supplement didactic teaching rather than replace it. Post-supplemental model was most preferred among students.

[1] CAL refers to computer-assisted learning.
[2] OPSE refers to Objective Structured Practical Exam.
[3] MCQ refers to multiple choice question.

Atray et al.<br><br>(2017)<br><br>(31)	India	Comparative study	Second-year medical students (total number of students not specified)<br><br>Demonstration 1: C[4] = 49, I[5] = 47, Demonstration 2: C = 49, I = 53, Demonstration 3: , C = 52, I = 55 Demonstration 4: C = 44, I = 45,	**C:** 4 demonstrations on the same drugs, conducted through conventional methods (animal experiments)<br><br>**I:** 4 animal simulator demonstrations on the effects of same drugs<br><br>All students were exposed to both modes of demonstrations.	Clinical pharmacology	**Objective:**<br>Four knowledge tests for each simulation (30 questions each, type not specified)<br><br>**Subjective:**<br>Likert-scale student evaluation of the learning experience	After each demonstration<br><br>Immediately after the intervention	Level 2b<br><br>Level 1b	Students who experienced the demonstrations through animal simulations scored significantly higher than those who learned through conventional demonstrations (p<0.05).<br><br>83% of respondents preferred simulation-based learning over conventional methods. Students found animal simulations more interesting and provided greater clarity.
Sikkens et al.<br><br>(2018)<br><br>(20)	Netherlands	Randomised controlled trial<br><br>(short-term effects)<br><br>Prospective controlled intervention study (long term effects)	480 fourth-year undergraduate medical students<br><br>Short term:<br>C = 56<br>I = 68<br><br>Long term:<br>C = 285<br>I = 71	**C**: No e-learning<br><br>**I:** interactive e-learning consisted of 8 clinical cases based on WHO[6] guide to good prescribing on the topic of antimicrobial prescribing. (Conducted over a 6-week period)	Prescribing skills	**Objective**:<br>Pre- and post-tests (57 MCQs) based on antimicrobial knowledge<br><br>OSCE[7] station where students needed to provide a written prescription for an infectious disease case<br><br>**Subjective**:<br>Student evaluation survey on the intervention (rated out of 10)	Before and immediately after the intervention<br><br>6 months after the intervention<br><br>Immediately after intervention	Level 2b<br><br>Level 2c<br><br>Level 1b	The short-term intervention group scored significantly higher on the post-intervention test (p = 0.032). Students from the long-term intervention group did significantly better in the test 6 months later (p= 0.031).<br>Students from the long-term intervention group scored significantly higher OSCE pass percentages compared to the control group (97% vs 86%, p< 0.05).<br><br>Medical students rated the intervention as instructive (scored 7.4/10). 77% of the students rated it as relevant whereas 55% of them rated it as too extensive. Students' self-perceived confidence in antimicrobial prescribing significantly increased (p=0.002).
Patel et al.<br><br>(2018)<br><br>(38)	India	Prospective single group pre-post intervention study	127 second-year undergraduate medical students	An online module with multimedia presentations including animations, pictures, case-based exercises on the topic of pharmacovigilance<br><br>(Students were given 14 days to use the e-learning resources before the post-intervention test)	Prescribing skills	**Objective**:<br>Pre- and post-intervention knowledge tests (SAQ[8]s and adverse drug reaction form filling exercise)<br><br>**Subjective**:<br>5-point Likert scale and open-ended student evaluation survey on the intervention	before and on completion of the intervention<br><br>On completion of the intervention	Level 2b<br><br>Level 1a, 1b	Students scored significantly higher in the post-intervention knowledge test for both SAQs and ADR form-filling exercise (p<0.01).<br><br>Overall, students appreciated the intervention and found it interesting and important. They also agreed that the intervention was informative and helpful in increasing their understanding of the topic.
Santhanalak shmi et al.<br><br>(2018)<br><br>(70)	India	Cross-sectional questionnaire based observational study	71 second-year undergraduate medical students	Animal experimental teaching followed by e-learning on the same topic (effect of diazepam on mice)	Clinical Pharmacology	**Objective**:<br>Knowledge test (MCQs) after animal experimental teaching and after CAL<br><br>**Subjective**:<br>5-point Likert scale student evaluation of the intervention	Immediately after the intervention<br><br>Immediately after the intervention	Level 2b<br><br>Level 1b	The average score of the same set of knowledge test improved after CAL compared to animal experimental teaching (82.4% vs 44.6%, no p value was provided).<br><br>Overall, most students accepted CAL as a teaching method for pharmacology and they found that CAL was more interesting, and it provided a better understanding of the topic than animal experimental teaching. However, many students agreed that CAL required more resources and computer expertise was required for CAL.

4 'C' is used as an abbreviation for control groups.
5 'I' is used an abbreviation for intervention groups.
6 WHO refers to World Health Organisation.
7 OSCE refers to Objective Structured Clinical Examination.
8 SAQ refers to short answer question.

Corbin et al.<br><br>(2018)<br><br>(55)	United States	Mixed methods evaluation study	21 first to fourth year undergraduate medical students	2-hour of SMARxT online video program on evidence-based prescribing with the aim to promote critical thinking on pharmaceutical marketing and to improve patient-provider relationship<br><br>**Other: online video program**	Prescribing skills	**Objective**:<br>Knowledge tests with MCQs<br><br>**Subjective**:<br>Likert scale student evaluation on learning experience	Before and immediately after intervention<br><br>Immediately after the intervention	Level 2b<br><br>Level 1b	Medical students performed significantly better in MCQ test after the intervention (37% increase with p<0.001).<br><br>76% of medical students agreed that they learnt new information from the intervention and 90% of them would recommend this intervention to others.
Badyal et al.<br><br>(2019)<br><br>(63)<br><br>**Other: immediate feedback**	India	Crossover study	48 second-year undergraduate medical students<br><br>**C** = 24<br>**I** = 24	**C**: Conventional teaching followed by online modules without feedback, on the topic of cardiovascular medications<br><br>**I:** conventional teaching followed by online interactive module with immediate feedback<br><br>Crossover was conducted on the topic of chemotherapy	Clinical pharmacology	**Objective**:<br>A knowledge test (SAQs and MCQs)<br><br>**Subjective**:<br>Likert scale student evaluation of the intervention and focus group interviews on the evaluation of the intervention	Immediately after the intervention<br><br>Immediately after the intervention and on completion of the intervention	Level 2b<br><br>Level 1a, 1b	There was no statistical difference between control and intervention groups in the knowledge test. (p=0.530)<br><br>Majority of the medical students agreed that the intervention improved their learning and knowledge, and it was stimulating and enjoyable. Students reported the intervention was motivating and useful.
Laks et al.<br><br>(2019)<br><br>(44)	Brazil	Before and after study	606 fifth and sixth-year undergraduate medical students	100-hour of web-based interactive e-learning activities including theoretical modules, exercises, and simulations, based on antimicrobial stewardship, accompanied by online tutorials and live discussion	Clinical pharmacology	**Objective**:<br>Knowledge tests (details not specified)<br><br>**Subjective**:<br>Student evaluation survey of the intervention	Before and immediately after the intervention<br><br>Immediately after the intervention	Level 2b<br><br>Level 1a	Medical students scored significantly higher in post-intervention test with p < 0.001.<br><br>69.5% of the medical students were satisfied or very satisfied with the course, whereas 30.2% of them expressed some degree of dissatisfaction. 69.5% of the students reported that they learned more than they expected.
Nalini et al.<br><br>(2019)<br><br>(74)	India	Post-intervention evaluation study	102 second-year undergraduate medical students<br><br>**C:** 51<br>**I:** 51	**C**: Students were provided with textbooks and underwent traditional learning on prescription writing<br>**I:** Students were provided with access to internet and underwent e-learning on prescription writing	Prescribing skills	**Objective**:<br>Prescription writing tests<br><br>**Subjective**: NA	Before and immediately after the intervention	Level 2c	Students from both groups performed significantly better in post-intervention prescription writing test. Students from the intervention group showed a more significant improvement (p<0.05).
Nettath<br><br>(2019)<br><br>(65)	India	Quasi-experimental study	80 second-year undergraduate medical students	**Lectured-based teaching**: 2 pharmacological topics were delivered by using lecture-based teaching<br><br>**CAL**: 2 different pharmacology topics were delivered using computer program which simulates drug actions to the same group of students	Clinical Pharmacology	**Objective**:<br>Knowledge tests (details not specified)<br><br>**Subjective**:<br>Student evaluation survey on the learning experience	Immediately after the intervention<br><br>Immediately after the intervention	Level 2b<br><br>Level 1b	Students scored significantly higher in the test followed by CAL compared to the lecture-based teaching (p<0.05).<br>Overall, there was no statistically significant difference between the mean score of students' perception towards e-learning and lecture-based learning in areas such as usefulness in scoring more marks and understanding the topics (p=0.094).
Elbeddini et al.<br><br>(2021)<br><br>(21)	Canada	Randomised controlled trial	16 third- or fourth-year post-graduate medical students<br><br>**C** = 6<br>**I** = 10	**C**: 5 reference articles on medication safety as reading materials<br><br>**I:** An online module on medication safety with interactive examples and case-based problem-solving<br><br>Both were conducted over a 2-week period.	Prescribing skills	**Objective**:<br>Knowledge tests (50 MCQs) on medication safety<br><br>**Subjective**: NA	Before and immediately after the intervention	Level 2b	Both control and intervention groups scored higher in the post-test, but the difference was more significant in the intervention group. Students from the Intervention group scored much higher than those from the control group in the post-test (difference was 13.4%, p<0.05).
Joseph et al.<br><br>(2021)<br><br>(29)	India	Comparative study	80 second-year undergraduate medical students<br><br>**C =** 40<br>**I =** 40	**C**: Students were given charts with graphs illustrating the effect of various drugs on animal tissue.<br>**I:** Computer simulation was provided to assist learning.<br><br>Both groups went through 1 topic in experimental pharmacology per week for 6 weeks.	Clinical pharmacology	**Objective**:<br>Test of academic performance<br><br>**Subjective**:<br>5-point Likert scale student evaluation of the intervention	Immediately after the intervention<br><br>Immediately after the intervention	Level 2b<br><br>Level 1b	The overall academic performance was higher for the simulation group compared to the chart group (p = 0.017).<br>95% of students in the simulation group reported that the intervention had helped them understand the subject compared to 70% in the chart-based learning group.

Riser et al.<br><br>(2021)<br><br>(45)	US	Before and after study	479 third-year undergraduate medical students	8-hour e-learning on medications for opioid use disorder	Prescribing skills	**Objective**:<br>Knowledge tests and prescription writing tests (details not specified)<br><br>**Subjective**:<br>Student evaluation survey on the intervention	Before and immediately after the intervention<br><br>Before and immediately after the intervention	Level 2b, 2c<br><br>Level 1b	There was a significant improvement in both pharmacology knowledge and prescribing skills on medications for opioid use disorder after the training (p<0.001).<br>Most students reported that this training was relevant to their future practice, and they plan to provide management of OUD in the future.
Mirza et al.<br><br>(2021)<br><br>(30)	India	Comparative study	109 second-year undergraduate medical students<br><br>C = 86 (total number of students who were in the control group)<br><br>I = 97 (total number of students who were in the intervention group)	**C:** Paper graphs and tracings were used to teach students about effect of drugs on rabbit eyes.<br><br>**I:** CAL was implemented to teach students about the effect of drugs on dog and rat blood pressure.<br><br>Crossover was conducted in the second session.	Clinical Pharmacology	**Objective**:<br>Knowledge test (MCQs)<br><br>**Subjective**:<br>5-point Likert scale student evaluation of the intervention	Immediately after the intervention<br><br>Immediately after the intervention	Level 2b<br><br>Level 1b	Students who were in the intervention group scored significantly higher in the knowledge test compared to those from the control group (p=0.005).<br>Majority of the students opined that the intervention was useful in helping them understand the topics in experimental pharmacology and apply this knowledge. They also agreed that they were more involved and motivated to learn with the implementation of the intervention.
**Simulation and Role-play**									
Lavanya et al.<br><br>(2016)<br><br>(86)	India	Post-intervention evaluation study	96 second-year undergraduate medical students	6 peer-role play sessions on medication communication with clinical. cases	Prescribing skills	**Objective**: NA<br><br>**Subjective**:<br>Student evaluation survey of the learning experience	Immediately after the intervention	Level 1a	84.3% of the medical students were satisfied with role play as a teaching and learning tool and 71.9% of them found the intervention useful when learning medication communication skills. 50% of the students felt competent at communicating drug names, dose, frequency, mechanism of action, purpose, and adverse effects.
Tayem et al.<br><br>(2016)<br><br>(87)	Bahrain	Post-intervention evaluation study	133 second-year undergraduate medical students	A workshop where clinical case scenarios on common cardiovascular diseases and prescriptions were discussed, and students were encouraged to perform role-play practices on the topic of medication communication with simulated patients (demonstration was given beforehand)	Prescribing skills	**Objective**:<br>OPSE on medication communication<br><br>**Subjective**:<br>5-point Likert scale student evaluation of the intervention<br><br>Open-ended student feedback (focus group interview)	At the end of the semester<br><br>Immediately after the intervention	Level 2c<br><br>Level 1b	Students who participated in the study scored significantly higher than those who did not participate (p=0.016).<br>Overall, students opined that the intervention was useful and their confidence and skills in medication communication improved.
Arcoraci et al.<br><br>(2019)<br><br>(32)	Italy	Comparative study	90 fifth-year undergraduate medical students	45-minute traditional lecture on inotropic agents, followed by simulation<br><br>3 groups: sham, low fidelity and high fidelity	Clinical pharmacology	**Objective**:<br>Knowledge tests (20 MCQs)<br><br>**Subjective**: NA	Before and immediately after the lecture; immediately after and 3 months after the simulation	Level 2b	The score improved across all three groups with p <0.01 immediately after lecture. High fidelity group scored significantly higher than sham and low fidelity groups immediately after simulation: (p<0.001). High fidelity group also significantly scored higher than sham and low fidelity groups 3 months post-intervention (p<0.001).
Hawley et al.<br><br>(2019)<br><br>(46)	United States	Before and after study	137 second-year medical students (undergraduate/post graduate not specified)	45-minute peer-role play simulation with one clinical case on reconciliation of the medications and identification of medication management errors.	Prescribing skills	**Objective**:<br>Knowledge tests (MCQs)<br><br>**Subjective**:<br>Likert scale student evaluation of the intervention	4 weeks before and 2 days after the intervention<br><br>Before and immediately after the intervention	Level 2b<br><br>level 1b	Medical students performed significantly better in post-test (p=0.009).<br><br>There was 41% increase in percentage of confident students after the intervention (p<0.001).
Kirsch et al.<br><br>(2019)<br><br>(104)	Germany	Post-intervention evaluation study	Undergraduate medical students (year level and number of participants not specified)	Students participated in case-based role-play practices with a focus on identification and evaluation of drug interactions and adverse drug reactions over a 1-week period.	Prescribing skills	**Objective**: NA<br><br>**Subjective**:<br>5-point Likert scale student evaluation and open-ended interview	Immediately after the intervention	Level 1a	Overall, students were acceptive of the intervention. Students mentioned phrases such as 'high clinical relevance' and 'relevant to medical practice'. Students

									also reported their learning deficits which included drug dosage, antibiotics, and drug interactions.
Nicolaou et al.<br><br>(2020)<br>(39)	Cyprus	Prospective cohort study	75 third- year undergraduate medical students<br><br>Cohort 1 = 31<br>Cohort 2 = 44	Both cohorts had 4 case-based tutorials, followed by 6 virtual patient simulation tutorials	Clinical pharmacology	**Objective**:<br>Cohort 1: Knowledge tests (30 & 60 MCQs respectively)<br><br>Cohort 2: Knowledge tests (100 MCQs)<br><br>**Subjective**:<br>Likert scale student evaluation of the intervention	Before the intervention (at mid-term) and at the end of the semester*<br>At the end of the semester*<br><br>Before the intervention and at the end of the semester	Level 2b<br><br>Level 2b<br><br>Level 1b	Medical students from cohort 1 performed significantly better in the final examination, compared with the midterm examination (p<0.05). Medical students from both cohorts performed significantly better on the questions related to teaching in the intervention compared to cased-based tutorials (p<0.05).<br><br>Medical students reported that the intervention facilitated their learning better than cased-based tutorials and it promoted both teamwork and independent learning.
**Interprofessional learning**									
Ward<br><br>(2016)<br>(76)	UK	Post-intervention evaluation study	106 final-year undergraduate medical students	3-hour interactive pharmacist-led workshop where students were divided into groups of 6 and focused on case-based prescribing scenarios e.g., medicines reconciliation	Prescribing skills	**Objective**: NA<br><br>**Subjective**:<br>Student evaluation of the intervention	On completion of the intervention	Level 1b	Overall, students found that the pharmacist-led teaching useful and they appreciated the interactive nature of the intervention.
Anderson et al.<br><br>2016<br>(56)	United Kingdom	Mixed-method evaluation study	294 final-year medical students	2-day practice-based learning with pharmacy students, assessing older in-patients with complex prescription regimens in a hospital setting	Prescribing skills	**Objective**: NA<br><br>**Subjective**:<br>Likert scale student evaluation of self-perceived ability<br><br>Qualitative open-ended feedback	Immediately after the intervention<br><br>Immediately after the intervention	Level 1b<br><br>Level 1b	Self- perceived ability improved significantly post-intervention. 94% of the medical students would recommend the course to others (p<0.001).<br><br>Themes included collaborative learning, enhanced competence and understanding of polypharmacy and challenges of working together in clinical practice.
Javadi et al.<br><br>(2017)<br>(47)	Iran	Before and after study	18 undergraduate medical students	18-hour interactive workshops conducted by clinical pharmacists, which consisted of 6 case-based didactic lectures and practical skill building training on pharmacotherapy	Prescribing skills	**Objective**:<br>Knowledge tests (details not specified)<br><br>**Subjective**:<br>Student evaluation survey on the learning experience (rated out of 10)	Before and immediately after the intervention<br><br>Immediately after intervention	Level 2b<br><br>Level 1a	Medical students preferred significantly better in the post-test (p value was not provided).<br><br>Medical students were highly satisfied with the intervention and the involvement of pharmacist in the intervention had the highest satisfaction rate (9.72/10).
Cooke et al.<br><br>(2017)<br>(78)	Ireland	Post-intervention evaluation study	9 undergraduate medical students	An interprofessional simulation workshop where medical students and pharmacy students engaged in patient consultation, prescribing, medication dispensing and medication counselling.	Prescribing skills	**Objective**: NA<br><br>**Subjective**: focus group interviews	On completion of the intervention	Level 1b	Medical students opined that the intervention improved their understanding in social and interpersonal aspects of prescribing and dispensing.
Kostas<br><br>(2018)<br>(77)	US	Post-intervention evaluation study	18 final-year postgraduate medical students	The 3-hour module consisted of a didactic lecture on medication history and review, a small interprofessional group discussion on three clinical cases and a large group presentation.	Prescribing skills	**Objective**: NA<br><br>**Subjective**:<br>Likert scale student evaluation of self-confidence in medication management competencies	On completion of the intervention	Level 1b	Overall, the intervention significantly improved students' abilities and confidence in 3 medication management competencies including the ability to identify high risk medications, document medications and explain the effect of physiological changes on drug dose and selection (p<0.05 for all three competencies).

Newby et al.<br><br>(2019)<br><br>(48)	Australia	Before and after study	16 final-year medical students	8-week pharmacist-led prescribing program, consisted of prescription writing practices, case-based tutorials, and exposure to the dispensing process in a hospital setting	Prescribing skills	**Objective**:<br>Prescribing tests<br><br>**Subjective**:<br>Student evaluation of self-perceived confidence<br><br>Student evaluation survey on the learning experience	Week 1 and week 8 of the intervention<br><br>Week 1 and week 8 of the intervention<br><br>Immediately after the intervention	Level 2c<br><br>Level 1b<br><br>Level 2a	No statistically significant improvement in appropriateness of prescribing among medical students (p = 0.087).<br>Medical students reported significant improvement in self-confidence in prescribing skills post-intervention (p<0.05).<br>All students agreed that the program improved their confidence and their awareness of good prescribing practice.
Guilding et al.<br><br>(2020)<br><br>(57)	United Kingdom	Mixed-method evaluation study	157 second year undergraduate medical students	1 introductory conference and 3 interprofessional workshops with case-based learning, video reflection and team-based simulation	Prescribing skills	**Objective**: NA<br><br>**Subjective**:<br>Likert scale student evaluation of the learning experience<br><br>Qualitative open-ended feedback	Immediately after the intervention<br><br>Immediately after the intervention	Level 1b<br><br>Level 1b	Most medical students agreed that interprofessional learning improved their communication skills, confidence and understanding of other professional expertise. Major reported learning gains included sepsis, antibiotics, how to use British National Formulary.
Allen et al.<br><br>(2020)<br><br>(49)	United States	Before and after study	First Intervention:<br><br>200 second-year postgraduate medical students<br><br>Second Intervention:<br>36 third-year postgraduate medical students	**First Intervention:** 3.5-hour workshop conducted by fourth-year pharmacy students where medical students work on prescribing exercises in small groups.<br><br>**Second Intervention:** 2-hour cased-based discussion led by fourth-year pharmacy students, followed by 2-hour practice session where medical students developed and presented case-based clinical questions	Prescribing skills	**Objective**:<br>OSCE stations on prescription writing<br><br>**Subjective**:<br>Likert-scale student evaluation of self-perceived confidence and learning experience<br><br>**Objective**: NA<br><br>**Subjective**:<br>Likert-scale student evaluation of self-perceived confidence and learning experience	At the end of the semester*<br><br>1 week before and 1 week after the intervention<br><br>Before and immediately after intervention	Level 2c<br><br>Level 1b<br><br>Level 1a, 1b	All medical students passed the OSCE assessment at the end of the semester.<br><br>Medical students felt significantly more confident in prescription writing post-intervention (p<0.001).<br><br>Medical students felt significantly more confident in counselling patients on the use of over-the-counter medications and making recommendations (p<0.001). The intervention was well received by both medical and pharmacy students.
**Case-based learning**									
Kumar et al.<br><br>(2016)<br><br>(79)	India	Post-intervention evaluation study	100 first- year undergraduate medical students	4 clinical topics were taught using case-based teaching method where students were divided into groups and encouraged to identify the learning issues and discuss them within and among groups with the presence of facilitators.	Clinical Pharmacology	**Objective**:<br>Knowledge tests (30 MCQs)<br><br>**Subjective**:<br>5-point Likert scale student evaluation of the intervention	Before and on completion of the intervention<br><br>On completion of the intervention	Level 2b<br><br>Level 1a, 1b	Students scored significantly higher across all four core topics in post-intervention test with p<0.00001.<br><br>Overall, students found the intervention helpful and better method of teaching compared to didactic lectures.
James et al.<br><br>(2016)<br><br>(80)	Bahrain	Post-intervention evaluation study	186 second-year undergraduate medical students<br><br>Small group = 116<br>large group = 70<br><br>(Students who attended the large group class also attended the small group class)	**Small group**: group discussion on clinical scenarios (based on WHO guide for good prescribing), prescribing skills, how to use British National Formulary<br><br>**Large group**: same process as above	Prescribing skills	**Objective**: NA<br><br>**Subjective**:<br>Likert scale student evaluation on the intervention (small vs large group)<br><br>Focus group interview on the evaluation of the intervention	Immediately after the intervention<br><br>On completion of the intervention	Level 1a<br><br>Level 1a	Most medical students perceived learning in small groups as a better approach to learning prescription writing.<br>Medical students reported that small group learning was better for learning overall.
Tichelaar et al.<br><br>(2016)<br><br>(27)	Netherlands	Randomised controlled minimal intervention study	251 second-year undergraduate medical students<br><br>**C**=100<br>**I** = 69 (WHO group)<br>**I** = 82 (SMART	A pharmacotherapy workshop where students were instructed to set treatment goals, prescribe medications, and determine how to monitor after treatment for four different patient cases with bronchial asthma	Prescribing skills	**Objective**:<br>Assessment of treatment goals, treatment choice and monitoring<br><br>**Subjective**: NA	During the intervention	Level 2b	Students from the SMART group scored significantly higher than WHO group and control group in treatment goal setting and monitoring (p<0.001 and p=0.004 respectively). There was no difference in average scores among three groups in treatment choice.

			group)	**C:** No specific instructions were given<br><br>**I** (WHO group): WHO 6-step method was given<br><br>**I** (SMART group): SMART criteria[9] for goal setting were given					
Sharma et al.<br><br>(2017)<br><br>(33)	India	Comparative study	135 second-year undergraduate medical students	**C:** Pure lecture-based teaching session given over the same period for all participants<br><br>**I:** Conventional lecture-based teaching sessions followed by 15-minute discussion on scenarios relevant to the topic of the lecture (over 3-month period)	Clinical Pharmacology	**Objective**:<br>A knowledge test (details not specified)<br><br>**Subjective**:<br>Likert scale and open-ended student evaluation on the intervention	On completion of the intervention<br><br>On completion of the intervention	Level 2b<br><br>Level 1a, 1b	Students scored higher on the topics covered in lectures with scenario -based teaching (62% vs 54%, no p value was given).<br>Overall, most students reported that scenario-based learning was interesting, motivating, and helpful in increasing their attention span, understanding of the concepts, and promoting critical thinking.
Palappallil et al.<br><br>(2019)<br><br>(66)	India	Quasi-experimental study	145 undergraduate medical students<br><br>*Year level not specified<br><br>Group A = 50<br>Group B = 46<br>Group C = 49	Group A: 1.5-hour task-based learning sessions where students were instructed to select a P-drug based on the clinical scenario and encouraged to complete the task in a small group setting, followed by a large group discussion<br><br>Group B: 40-minute case-based learning session where students were instructed to select appropriate P-drugs in the context of doctor-patient role-plays<br><br>Group C: 30-minute didactic learning sessions where conventional lectures were delivered to students on the topics of P-drug selection<br><br>Each group received 6 sessions on the same topics of P-drug selection e.g. bronchial asthma, type 2 diabetes, and angina	Prescribing skills	**Objective**:<br>A knowledge test (filling in the blanks, MCQs and clinical case-based tasks)<br><br>**Subjective**:<br>5-point Likert scale student evaluation of the intervention	Immediately after the intervention<br><br>Immediately after the intervention	Level 2b<br><br>Level 1a, 1b	The mean scores among the students from task-based learning group and didactic learning were significantly higher than those from group case-based learning (p<0.001). Task-based learning group performed significantly better in questions related to p-drug selection and prescription for peptic ulcer, listing steps of P-drug selection compared to the other two groups (p<0.001).<br>Case-based learning was most interesting and well-liked by students compared to task-based learning and didactic learning (p<0.001). Overall, most students from all three groups opined that the interventions were useful in gaining knowledge and skills to select P-drug for patients.
Field<br><br>(2019)<br><br>(50)	United Kingdom	Before and after study	201 final-year medical students	Four 1.5-hour team-based revision tutorials based on Prescribing Safety Assessment (assess the outcomes published in Tomorrow's Doctors)	Prescribing skills	**Objective**: NA<br><br>**Subjective**:<br>Likert scale student evaluation on self-perceived confidence in prescribing and self-attitudes towards teamwork pre-and post- intervention	Before and immediately after intervention	Level 1b	Medical students felt significantly more confident in prescription post-intervention (p<0.0001). Most medical students agreed that intervention was helpful for preparation for the Prescribing Safety Assessment and internship.
Hasamnis et al.<br><br>(2019)<br><br>(81)	Malaysia	Post-intervention evaluation study	80 second-year undergraduate medical students	40-minute tutorial on 4 case-based clinical scenarios involving small group discussion and case presentation	Clinical pharmacology	**Objective**: NA<br><br>**Subjective**:<br>Student evaluation on the intervention based on DREEM[10] questionnaire	Immediately after the intervention	Level 1a	The average DREEM score was 155/200 which indicated "excellence in learning pharmacology through CBL session" and majority of the medical students enjoyed learning and were satisfied with the understanding of the content.

9 SMART criteria have been used to optimise treatment goal setting and SMART stands for Specific, Measurable, Acceptable, Realistic and Time-bound. [27]
10 The DREEM is a validated measurement tool developed to assess the educational environment of health professionals who are under training.[105]

Mozeika et al.<br><br>(2020)<br><br>[105]	US	Post-intervention evaluation study	180 first-year undergraduate medical students	4 small group-based tutorials, where students participated in the group discussions on clinical cases and quiz with access to reading materials	Clinical pharmacology	**Objective**:<br>Group quiz (19 MCQs)<br><br>**Subjective**:<br>Likert scale student evaluation on the intervention	During the intervention<br><br>Immediately after the intervention	Level 2b<br><br>Level 1a	The average score of the group quizzes was 86%.<br><br>Overall, medical students reported the intervention provided worthwhile information and the group learning was productive.
Kaur et al.<br><br>(2020)<br><br>[60]	India	Crossover study	94 second-year undergraduate medical students<br><br>**C** = 47<br>**I** = 47	**C**: traditional didactic lecture on acid peptic disorder and treatment<br><br>**I:** 2-hour cased-based learning based on the same topic in a large group setting<br><br>Crossover was conducted on the topic of tuberculosis medications	Clinical pharmacology	**Objective**:<br>A knowledge test consisting of a mixture of essay questions, SAQs, and MCQs<br><br>**Subjective**:<br>Likert-Scale student evaluation on the intervention	2 weeks after the intervention<br><br>Immediately after the intervention	Level 2b<br><br>Level 1b	There was no statistical difference in the post-intervention test scores between the two groups (p = 0.98) but the medical students from the intervention group did better in questions involving critical thinking whereas control group did better in knowledge-based questions (p<0.001).<br>Most of the students reported that the intervention was useful, and it improved their ability to apply theory in a clinical setting and skills in identifying potential medication-related difficulties of patients.
Brinkman et al.<br><br>(2021)<br><br>[34]	Portugal	Comparative study	90 final-year medical students<br><br>**C** = 36<br>**I** = 54	**C:** Traditional methods of teaching pharmacology through lectures and written assessments<br><br>**I:** Integrated teaching of pharmacology with pathophysiology, microbiology, and neurosciences in second and third years of the medical course, followed by a problem-based learning course in the final year with students working through patient cases under the supervision of clinical pharmacologists	Prescribing skills	**Objective:**<br>Web-based assessment tool consisting of 24 MCQs and five clinical case scenarios requiring written prescriptions<br><br>**Subjective:**<br>Student evaluation survey on the learning experience	At the end of final year<br><br>At the end of final year	Level 2b, 2c<br><br>Level 1b	Students in the intervention group scored significantly better than the control group (p=0.002) and made fewer inappropriate therapy choices (p=0.023).<br><br>Students in the intervention group were significantly more confident in their prescribing skills.
**Peer-led learning**									
Sukhlecha et al.<br><br>(2016)<br><br>[22]	India	Randomised controlled trial	141 second-year undergraduate medical students<br><br>**C** = 71<br>**I** = 70	**C:** 3 60 to 80-minute didactic tutorials<br><br>**I:** 3 60 to 80-minute SLOT[11] where students were divided into groups and prepared 5 MCQs related to chemotherapy. The MCQs were presented during the class and all other groups all participated in answering the questions and group discussion.	Clinical pharmacology	**Objective**:<br>Knowledge tests consisting of 20 SAQs<br><br>**Subjective**:<br>Likert-Scale student evaluation on the intervention	Before and on completion of the intervention<br><br>Immediately after the intervention	Level 2b<br><br>Level 1a	There was 31% of improvement in post-test in the intervention group compared to 11% in the control group (no p value provided).<br><br>78% of the medical students reported they would prefer SLOT to conventional tutorials and 76% of them agreed that SLOT stimulated their interest and improved their self-learning skills.
Arora et al.<br><br>(2016)<br><br>[23]	India	Randomised controlled trial	173 second-year undergraduate medical students<br><br>**C** = 87<br>**I**= 86	**C**: 5 didactic tutorials led by tutors on the topics such as sedatives, analgesics etc.<br><br>**I:** 5 SLOTs where students were divided into groups and prepared 4-5 MCQs related to the same topics as the control group. The MCQs were presented during the class and all other groups all participated in answering the questions and group discussion.	Clinical pharmacology	**Objective**:<br>A knowledge test consisting of essay questions, SAQs and MCQs<br><br>**Subjective**:<br>Likert-scale student evaluation on the intervention	Immediately after the intervention<br><br>Immediately after the intervention	Level 2b<br><br>Level 1a	There was no statistical difference between average scores of two groups (p=0.391) but the pass rate was significantly higher in the intervention group (p<0.05).<br><br>64% of the students from the intervention group agreed that they learnt more from SLOT than didactic teaching. 87% of them reported SLOT was stimulating whilst some believed SLOT was time consuming and required a lot of preparation.

[11] SLOT refers to student-led objective tutorials.

Shenoy et al.<br><br>(2021)<br><br>(35)	India	Comparative study	203 second-year undergraduate medical students	**SLOT group**: 1-hour SLOT tutorial on the topics of NSAIDs, medications used in rheumatoid arthritis and gout<br><br>**Crossword puzzle group**: 1-hour tutorial for students to solve crossword puzzles on the same topics	Clinical Pharmacology	**Objective**:<br>A knowledge test consisting of MCQs and SAQs<br><br>**Subjective**:<br>5-point Likert scale and open-ended student evaluation of the intervention	One week after the intervention<br><br><br>Immediately after intervention	Level 2b<br><br><br><br>Level 1a, 1b	Students from the SLOT group performed significantly better in the post-intervention knowledge test compared to those from the crossword puzzle group (p<0.001).<br>Students from the SLOT group reported that the intervention was innovative and creative. SLOT improved their in-depth learning and promoted their critical thinking; however, it was also considered as time-consuming. Students from the crossword puzzle group opined that the intervention was innovative and fun. It helped with memorisation of drug names but lacked in-depth learning experience.
**Experiential learning**									
Thenrajan et al.<br><br>(2016)<br><br>(36)	Netherlands	Comparative study	50 second-year undergraduate medical students<br><br>**C** = 25<br>**I** = 25	WHO guidelines and prescribing writing introduction were given<br><br>**C**: Case-based learning<br>**I:** Patient-based learning based on the same case scenarios<br><br>Followed by 2-day self-study for both groups	Prescribing skills	**Objective**:<br>A prescription writing test<br><br>**Subjective**:<br>Open-ended student feedback	2-days after in the intervention<br><br>2-days after in the intervention	Level 2c<br><br><br>Level 1a, 2a	Medical students from the intervention group did significantly better than those from the control group in the prescription writing test (p<0.001).<br>Students perceived that the intervention was more interesting and motivating and they felt more responsible and empathic toward patients.
Singh et al.<br><br>(2017)<br><br>(61)	India	Crossover study	60 undergraduate medical students<br><br>Group A = 20<br>Group B = 20<br>Group C = 20<br><br>(Year level not specified)	A didactic lecture on cardiovascular pharmacology was given to all students<br><br>Phase I: bedside teaching for group A, routine tutorial classes for group B & C, followed by a knowledge test for all three groups<br><br>Phase II: bedside teaching for group B, routine tutorial classes for group A & C, followed by a knowledge test for all three groups<br><br>Phase III: no intervention, a knowledge test for all three groups	Clinical Pharmacology	**Objective**:<br>Pre-intervention knowledge test<br><br>Post phase I & II knowledge test<br><br>Post phase III knowledge test<br><br>(30MCQs each)<br><br>**Subjective**:<br>Student evaluation survey on the intervention	Before the intervention<br>On completion of the intervention<br>One month after phase II<br><br><br><br>On completion of the intervention	Level 2b<br><br><br><br><br><br><br>Level 1b	During phase I, group A scored significantly higher in the knowledge test than group B and C (p<0.05) and during phase II, group B scored significantly higher than control group C (p<0.05) but showed no difference when compared to group A. During phase III, group C performed significantly poorer compared to group A and C (p<0.05).<br><br>Most students agreed that the lecture remains the best method for teaching pharmacology, however, more than half of the students from group A and B opined that the bed side teaching improved their learning and memorisation of medications.
Kinston et al.<br><br>(2019)<br><br>(82)	United Kingdom	Post-intervention evaluation study	108 final-year undergraduate medical students	Practices of prescription writing for in-patients during 15-week clinical placement	Prescribing skills	**Objective**: NA<br><br>**Subjective**:<br>Student evaluation on the educational intervention	Immediately after the intervention	Level 1b	All medical students agreed that the intervention was helpful in aiding their learning about prescribing, and 97.2% of them agreed that their confidence in prescribing had increased post-intervention.
Gupta et al.<br><br>(2019)<br><br>(83)	United Kingdom	Post-intervention evaluation study	30 fourth and fifth-year undergraduate medical students	2-week of real-patient medication-related consultation by using patient education checklist	Prescribing skills	**Objective**: NA<br><br>**Subjective**:<br>Open-ended student feedback	Immediately after the intervention	Level 1b	Most medical students reported that they felt more confident about counselling patients on medication-related issues and their pharmacology knowledge. 83% of the students found patient education checklist useful.
Jose et al.<br><br>(2021)<br><br>(84)	India	Post-intervention evaluation study	130 second-year undergraduate medical students	2-hour session consisting of a lecture on the pharmacology of medications for diabetes, lipid dysfunction, angina, and thyroid disorders followed by completion of an online form by students through involving a patient/relative/friend who had the specific condition.	Prescribing skills	**Objective**: N/A<br><br>**Subjective**:<br>5-point Likert scale student evaluation of the intervention	Immediately after the intervention	Level 1a, 1b	81% (81/100) of students felt happy to be part of the project. 63% reported developing self-confidence in learning the subject with a median score of 4. 76% reported usefulness in understanding theoretical contents in pharmacology.
Reumerman	Netherlands	Prospective	80 first to sixth-year	**C**: no intervention	Prescribing	**Objective**:			

et al.<br><br>(2021)<br><br>(40)		observational study	undergraduate medical students<br><br>C = 39<br>I = 41	**I:** Practices on identifying patients with potential adverse drug reactions on the ward<br><br>Conducted over 12-months period	skills	Knowledge tests (16 questions, the type of questions was not specified)<br><br>**Subjective**:<br>5-point Likert scale student evaluation of the intervention	4 weeks before and 1week after the intervention<br><br>4 weeks before and 1week after the intervention	Level 2b<br><br>Level 2a	Medical students from the intervention group did significantly better than those from the control group in the knowledge test (p< 0.05). They were also significantly more skilled at detecting adverse drug reactions compared to those from the control group (p<0.05).<br>There was a considerable improvement in awareness and understanding of the importance of adverse drug reaction recognition (p=0.031, p<0.001).
**Game-based learning**									
Karbownik et al.<br><br>(2016)<br><br>(26)	Poland	Randomised controlled trial	124 third-year postgraduate medical students<br><br>C = 61<br>I = 63	**C**: 75-minute lecture-based seminar on antimicrobial drugs<br><br>**I:** 75-minute board game-based learning on antimicrobial drugs	Clinical pharmacology	**Objective**:<br>Knowledge tests (20 yes/no questions)<br><br>Final examination in pharmacology on antimicrobials (6-12 MCQs)<br><br>**Subjective**:<br>4-point Likert scale student evaluation of the intervention	Before and immediately after the intervention<br><br>14 days (median) after the intervention (range: 3-77 days)<br><br>Immediately after the intervention	Level 2b<br><br>Level 2b<br><br>Level 1a	There was a significant increase in short-term knowledge among medical students from both groups after the intervention (both p<0.0001) but there was no significant difference between two groups (p=0.2527).<br>Medical students from the Intervention group scored significantly higher than the control group in the final exam (p=0.007).<br><br>Overall, the intervention was better perceived than the control by the medical students.
Aynsley et al.<br><br>(2018)<br><br>(58)	United Kingdom	Mixed methods evaluation study	125 second-year undergraduate medical students	90 min Braincept game-based learning	Clinical pharmacology	**Objective**:<br>Knowledge tests (3 SAQs)<br><br>**Subjective**:<br>Likert scale student evaluation on the learning experience	Before, immediately after and 3 days after the intervention<br><br>Before and immediately after the intervention	Level 2b<br><br>Level 1b	Medical students improved their learning performance immediately and 3-day post-intervention, 45% and 28% increase respectively (no p-value was provided).<br><br>There was dramatic increase in self-perceived confidence among medical students post intervention (86% increase, no p-value was provided). 98% of medical students reported that the intervention was a good approach to pharmacology learning.
Lee et al<br><br>(2019)<br><br>(88)	US	Post-intervention evaluation study	44 second-year postgraduate medical students	2 electronic educational games on clinical pharmacological topics, designed for individual or group play, one with limited cue recall and open-ended questions, the other with MCQs in a trivial format	Clinical Pharmacology	**Objective**:<br>Knowledge tests (12 MCQs)<br><br>**Subjective**:<br>Likert scale Student evaluation survey on the intervention	Before and immediately after the intervention<br><br>Immediately after the intervention	Level 2b<br><br>Level 1a, 1b	There was a significant increase in post-intervention knowledge test scores among medical students (p=0.03) which indicated that the intervention improved short-term performance.<br>Most students reported that the games were engaging, efficient and useful in reinforcing second year knowledge.
Davies<br><br>(2020)<br><br>(51)	United Kingdom	Before and after study	60 first-year post-graduate medical students	A single 10-minute antibiotic-themed game in the style of 'Top Trumps'®. Success at the game relies on remembering the relative scores of various features of each item.	Clinical pharmacology	**Objective**:<br>Knowledge tests (SAQs)<br><br>**Subjective**:<br>Binary (yes/no) student evaluation of the intervention	Before and immediately after the intervention<br><br>Immediately after the intervention	Level 2b<br><br>Level 1b	There was no difference in test scores before and after playing with the cards.<br><br>23/36 (64%) reported finding the game useful for learning.
Gudadappan avar et al.<br><br>(2021)<br><br>(24)	India	Randomised controlled trial	98 undergraduate medical students (year not specified)<br><br>**C** = 50<br>**I** = 48	**C:** Didactic learning tutorials<br><br>**I:** Game-based learning tutorials in small groups (12 students per group)<br><br>Conducted over 5-month period	Clinical pharmacology	**Objective**:<br>Knowledge tests (40 MCQs)<br><br>**Subjective**:<br>Likert scale student evaluation on the learning experience and usefulness of the intervention with yes/no questions	Before and on completion of the intervention*<br><br>On completion of the intervention	Level 2b<br><br>Level 1a, 1b	There was no difference in pre-test. Medical students from the intervention group scored higher in the post-test (p<0.001) and both groups improved their knowledge (p<0.001).<br>Medical students from the intervention group enjoyed more as the intervention made the learning more interactive, collaborative and they also felt more confident. Medical students from the intervention group perceived the intervention more useful.
**Multicomponent**									
Wallace et al.<br><br>(2016)<br><br>(42)	Australia	Prospective cohort study	163 second-year undergraduate medical students	2-hour workshop consisting of pre-readings, small group discussions on prescription writing, dose calculation and medication administration, followed by a hurdle practice test with 15 SAQs (repeated until the student	Prescribing skills	**Objective**:<br>Prescription writing tests<br><br>**Subjective**:<br>Likert scale student evaluation on the	Immediately after and 4 years after the intervention<br><br>4 years after the	Level 2c<br><br>Level 1b	There was no statistical difference between the scores of pre-and post-tests indicating that the knowledge was retained after 4 years (p=0.57).<br><br>About 60% of the medical students reported as feeling

				was deemed competent).		intervention	intervention		confident in medication calculations and in drawing up parental medications. 41% of the students used the skills that they learnt from the intervention in clinical placement.
Nori et al.<br><br>(2017)<br><br>(67)	US	Quasi-experimental before and after study	549 second-year postgraduate medical students	One lecture on antimicrobial stewardship and infection prevention, accompanied by a printed antibiogram (2014) and a smart phone application called "Appropriate Use" (2015, 2016)<br><br>The intervention was implemented over a 3-year span.	Clinical Pharmacology	**Objective**:<br>Knowledge tests<br><br>**Subjective**:<br>Student evaluation survey of the intervention	Before and on completion of the intervention<br><br>Before and on completion of the intervention	Level 2b<br><br>Level 1b	Students performed significantly better for questions involving antibiogram use (p<0.05), however, there was no significant improvement in questions related to antibiotic use (p=0.64).<br>About 70% of students agreed that they felt more comfortable at prescribing antibiotics in comparison to 40% prior to the intervention (<0.05). Up to 69% of students would prefer using the application as the source of antibiotic information.
Raghu et al.<br><br>(2017)<br><br>(71)	India	Cross-sectional observational study	117 second-year undergraduate medical students	A rational prescribing lecture and a group discussion on prescriptions written by students based on clinical case scenarios, followed by feedback from the facilitators	Prescribing skills	**Objective**:<br>Prescription writing tests<br><br>**Subjective**: NA	Before and during the intervention	Level 2c	After the intervention, there was a significant decrease in the percentage of errors e.g. drug choice, patient information and prescriber information (p<0.05).
Sırrı Bilge et al.<br><br>(2017)<br><br>(89)	Turkey	Post-intervention evaluation study	400 fourth-year medical students (undergraduate/post graduate – not specified)	5-day program consisting of 5 different lecture presentations, prescribing practices (based on WHO guide on good prescribing) and case discussion	Prescribing skills	**Objective**:<br>OSCE with simulated patients<br><br>**Subjective**:<br>Student evaluation of the intervention with yes/no" questions	Immediately after the intervention<br><br>Immediately after the intervention	Level 2c<br><br>Level 1a, 1b	The overall average score for the exam was 94.8 (the full score was not specified).<br><br>The intervention was well received by the medical students. Most medical students rated the components of the intervention as "positive". Most students agreed that the educational intervention is useful for improvement of their prescribing knowledge and skills.
Hauser et al.<br><br>(2017)<br><br>(90)	Germany	Post-intervention evaluation study	40 third to fifth-year medical students<br><br>(undergraduate/post graduate not specified)	2 problem-based learning tutorials, followed by 1 workshop on development of prescription talk guide and prescription talk simulation. An online-based learning platform was provided for students to access relevant publications	Prescribing skills	**Objective**: NA<br><br>**Subjective**:<br>Student evaluation survey on the learning experience	On completion of the intervention	Level 2a	Overall, medical students responded positively and there was an enhanced awareness of the importance of medical communication.
Eriksson et al.<br><br>(2018)<br><br>(91)	Sweden	Post-intervention evaluation study	195 medical students<br><br>C: 101<br>I: 137	**C:** Clinical pharmacology course before modification which consists of 5 days of lectures and seminars, case-based discussion<br><br>**I:** Modified course consisting of ward-based education, lectures, patient case-based discussion, clarified learning objectives and a list of common drugs is given to the students	Prescribing skills	**Objective**: NA<br><br>**Subjective**:<br>Likert scale student self-evaluation of their own prescribing skills	On completion of the pharmacology course	Level 1b	Students in the intervention group reported higher confidence in basic prescribing skills including writing medication discharge summaries and performing medication reviews (p=0.008, p=0.024 respectively). There was also a positive correlation between the number of medications reviews or discharge summaries performed and level of confidence in prescribing skills.
Oldfield<br><br>(2020)<br><br>(52)	US	Before and after study	75 postgraduate medical students<br><br>*Year level not specified	A 75-minute workshop consisting of a group discussion on opioid overdose, a didactic session on harm reduction and demonstrations on how to use naloxone	Clinical Pharmacology	**Objective**:<br>Knowledge tests (38 True or false & MCQs)<br><br>**Subjective**:<br>5-point Likert Scale student evaluation of the intervention	6 weeks before and 6 weeks after the intervention<br><br>Before and 6 weeks after the intervention	Level 2b<br><br>Level 1b, 2a	Students performed significantly better in the knowledge test 6 weeks after the intervention (p<0.001).<br><br>There was a significant improvement in preparedness to address opioid overdoses among students (p<0.001) and moderate improvement in attitudes towards patients with substance use disorders (p=0.04).

Jain et al.<br>(2020)<br>(92)	India	Post-intervention evaluation study	105 second-year undergraduate medical students	40-min interactive lecture on arrhythmia management followed by small group development of concept maps, facilitated by faculty members.	Clinical pharmacology	**Objective**: NA<br>**Subjective**:<br>Student evaluation on enjoyment of intervention and usefulness	Immediately after the intervention	Level 1a, 1b	81/104 (77.9%) found constructing enjoyable and 91/104 (87.5%) found the concept mapping useful as a teaching strategy in pharmacology.
Mchugh et al.<br>(2021)<br>(93)	US	Post-intervention evaluation study	365 first-year postgraduate medical students<br>(Students were recruited from 2014-2018 cohorts)	15 online modules on pharmacodynamics and pharmacokinetics, followed by lectures and online group assignments	Clinical Pharmacology	**Objective**:<br>15 formative group assignments and summative individual assignments<br>A knowledge test (MCQs)<br>**Subjective**:<br>4-point Likert Scale student evaluation of the intervention	During the intervention<br>6 weeks after the intervention<br>On completion of the intervention	Level 2b<br>Level 2b<br>Level 1b	For group assignments, 7.8-15.6% of students failed due to late submission whereas all students passed the final individual assessment.<br>All students were deemed to be competent based on the test results.<br>The majority of students agreed that the intervention was extremely or somewhat valuable in helping them achieve the learning objectives.
Loya et al.<br>(2021)<br>(94)	US	Post-intervention evaluation study	68 third-year postgraduate medical students	A 16-week selective in pharmacotherapeutics in Primary Care where a wide range of learning strategies were employed including case-based learning, didactic lectures, experiential learning, and online readings.	Prescribing skills	**Objective**:<br>Knowledge tests (17 MCQs) and 4 case-based assessments (dose calculations, pharmacotherapy monitoring strategies etc.)<br>**Subjective**:<br>4-point Likert Scale student evaluation of the intervention	Before and immediately after the intervention<br>Before and immediately after the intervention	Level 2b, 2c<br>Level 1b	Students scored significantly higher in both post-intervention knowledge tests and case-based assessments with p<0.001.<br>Overall, students felt more confident in taking a medication history, evaluating medication regimen, and identifying adverse drug events. They also felt more confident in working collaboratively with our healthcare professionals.
Nayak et al.<br>(2021)<br>(53)	India	Before and after study	60 undergraduate medical students	2 workshops on prescribing competency as per WHO Guide to Good Prescribing, consisting of group discussion, prescribing practice, and role play<br>(Duration of workshops not specified)	Prescribing skills	**Objective**:<br>OPSEs on prescription writing, critical appraisal of a given prescription and medication communication<br>**Subjective**:<br>Likert scale student evaluation on the intervention and open-ended feedback	Before and immediately after the intervention<br>Immediately after the intervention	Level 2c<br>Level 1a	There was significant improvement in the post-intervention OPSEs on prescription writing (p<0.001).<br>Most medical students found the intervention inspiring and motivating.
**Other**									
Kalra et al.<br>(2016)<br>(95)	India	Post-intervention evaluation study	120 undergraduate medical students<br>(Year level not specified)	Poem writing on the topic of asthma medications	Clinical pharmacology	**Objective**: NA<br>**Subjective**:<br>Open-ended student feedback	On completion of the intervention	Level 1a	Most medical students perceived the intervention as positive and enjoyable whilst some found the intervention childish, exhaustive, and average.
Yadav et al.<br>(2016)<br>(62)	India	Crossover study	165 second year undergraduate medical students	**C**: Conventional teaching where students were given lectures in separate disciplines, on the topic of epilepsy<br>**I:** Integrated teaching on the topic of epilepsy<br>Crossover was conducted on the topic of tuberculosis (all participants were given a sensitisation workshop and reading materials on integrated teaching beforehand)	Clinical Pharmacology	**Objective**:<br>pre-and post-intervention knowledge tests<br>**Subjective**:<br>Likert scale student evaluation on the intervention	Before and on completion of the intervention<br>On completion of the intervention	Level 2b<br>Level 1b	Both groups showed an improvement in post-intervention knowledge tests. The intervention group scored higher than the conventional group on the topic of epilepsy whereas the conventional group did better than the intervention group on tuberculosis (no p value provided).<br>Overall, students reported that the integrated teaching was useful in learning a topic and reducing time for self-study but not so strongly about increasing self-confidence towards learning.
Ahsan et al.<br>(2016)<br>(25)	India	Randomised controlled trial	150 second-year undergraduate medical students<br>**C**= 75	**C**: no intervention<br>**I:** clinical case practice given 5 days prior to the lecture	Clinical pharmacology	**Objective**:<br>Knowledge tests (30 MCQs)<br>**Subjective**:<br>Student evaluation survey of the	2 days after the intervention<br>Immediately after the	Level 2b<br>Level 1a	Medical students from the intervention group did significantly better than the control group (p<0.01) in the knowledge assessment.<br>Most students from both groups found the learning

			**I** = 75	1-hour lecture on drug therapy of peptic ulcer delivered to both groups		intervention	intervention		experience was positive. Higher percentage of students from the intervention group felt confident in answering questions related to drugs used for peptic ulcer.
Ying et al.<br><br>(2017)<br><br>(68)	Malaysia	Quasi-experimental study	222 third year undergraduate medical students<br><br>**C** = 100<br>**I** = 122	48-hour lecture pharmacology course, accompanied by self-study<br><br>**C:** Regular study strategies<br><br>**I:** Mind maps as one of the study strategies (students were introduced to mind mapping beforehand)<br><br>Regular study strategies were not specified in the study.	Clinical Pharmacology	**Objective**:<br>Knowledge test (final course exam with SAQs, MCQs, filling in the blank and case analysis)<br><br>**Subjective**:<br>Student evaluation of the intervention	At the end of the semester (no specific timing was given)<br><br>At the end of the semester (no specific timing was given)	Level 2b<br><br>Level 1b	The intervention group performed significantly better than the control group int he post-intervention knowledge tests across all four domains and in average (p<0.01).<br><br>Overall, students from the intervention group reported that mind maps were useful in facilitating their learning in pharmacology. More than 85% of the students agreed that they would use mind maps as an alternative study method.
Hasamnis<br><br>(2017)<br><br>(106)	Malaysia	Post-intervention evaluation study	first-year undergraduate medical students	1-hour small group worksheet-based discussion followed by interactive discussion-cum-feedback by the lecturer on the topic of anti-arrhythmic drugs	Clinical pharmacology	**Objective**: NA<br><br>**Subjective**:<br>Student evaluation on the intervention based on DREEM questionnaire	Immediately after the intervention	Level 1a	The average DREEM score was 139.7/200 indicating that students had “more positive than negative feelings” and enjoyed learning antiarrhythmic drugs in small-group discussion.
Parmar<br><br>(2018)<br><br>(41)	India	Prospective interventional study	173 second-year undergraduate medical students	A museum for students where medications on autonomic nervous system, cardiovascular system and chemotherapy were displayed. The relevant lectures were delivered prior to the visits.<br><br>*Visits to the museum were mandatory	Clinical Pharmacology	**Objective**:<br>Knowledge tests (21 MCQs)<br><br>**Subjective**:<br>3-point Likert scale and open-ended student evaluation survey of the intervention	Before and on completion of the intervention<br><br>On completion of the intervention	Level 2b<br><br>Level 1a, 1b	Students scored significantly higher in the post-intervention knowledge test (p<0.001).<br><br>Around half of the students found the information displayed in the museum was helpful and inspiring. Less than 50% of them agreed that the information was adequate or brought clarity. However, more than 75% of students opined that the museum was attractive.
Decloedt et al.<br><br>(2019)<br><br>(96)	South Africa	Post-intervention evaluation study	545 third-year undergraduate medical students	Creation of 2-minute video on pharmacotherapy for patient education	Clinical pharmacology	**Objective**: NA<br><br>**Subjective**:<br>Open-ended student feedback	On completion of the intervention	Level 1a, 1b	Medical students reported that the intervention provided them with enjoyable learning experience, but it was time consuming and had limited value of therapeutics education.
Atif Beg et al.<br><br>(2020)<br><br>(59)	India	Mixed methods evaluation study	102 second-year medical students	Pre-reading materials were provided 5-7 days prior to each of 4 lectures on 4 different classes of drugs. 20 minutes during each lecture was dedicated to interactive discussion of the pre-reading material. No pre-reading material was given for 4 other lectures on other different classes of drugs	Clinical pharmacology	**Objective:**<br>A knowledge test on both the content with pre-reading and the one without (MCQs and SAQs)<br><br>**Subjective:**<br>Likert scale student evaluation on the learning experience	Immediately after the intervention<br><br>Immediately after the intervention	Level 2b<br><br>Level 1b	Statistically significant difference in favour of content where pre-reading material was provided (p=0.01).<br><br>Majority of the students found pre-readings improved their understanding of material, interest and made the lectures more interactive. A third of students did find the intervention burdensome to their existing workload.
Gossell-Williams et al.<br><br>(2020)<br><br>(107)	Jamaica	Post-intervention evaluation study	10 first- and second-year undergraduate medical students	5-session program (14 hours in total) with an introductory lecture followed by student oral and poster presentation based on literature searches on a single adverse drug reaction.	Clinical pharmacology	**Objective**: N/A<br><br>**Subjective**:<br>Student evaluation of the usefulness scale 1=poor,10= excellent) and self-perceived confidence (yes/no)	Immediately after the intervention	Level 1b	Students reported the intervention to be useful (mean score 9.1) and 8/10 students reported more confidence with pharmacology knowledge.

Purohit et al.<br><br>(2020)<br>[97]	India	Post-intervention evaluation study	112 second-year undergraduate medical students	2.5-hour activity where autobiography of ten medications were displayed and students were encouraged to identify the medication in the format of group discussion	Clinical Pharmacology	**Objective**: NA<br><br>**Subjective**:<br>5-point Likert scale and open-ended student evaluation survey of the intervention	 <br><br>Immediately after the intervention	 <br><br>Level 1a, 1b	 <br><br>Most of the students reported that the intervention was interesting, enjoyable, and engaging. About 75% of the students opined that the intervention was helpful in information recall and knowledge retention. However, some students mentioned that the intervention requires creativity and they found it difficult to learn pharmacology in this regard.
Cambra-Badii et al.<br><br>(2020)<br>[54]	Spain	Before and after study	Subset of 170 medical students in 237 students enrolled in a pharmacology course<br><br>Third-year: 67<br>Fifth-year: 59<br>Sixth-year: 44	50-minute clip of different scenes from the film "150 Milligrams" followed by 30-minute discussion on the aspects of the film	Clinical pharmacology	**Objective:**<br>Knowledge tests (10 MCQs)<br><br>**Subjective:**<br>Likert-scale student evaluation on the learning experience	 Immediately before and immediately after the intervention<br><br>Immediately after the intervention	 Level 2b<br><br>Level 1b	 Student knowledge significantly improved following the intervention. Third-year medical students made the greatest gain (p<0.001), followed by fifth-years (p>0.001) then sixth-years (p=0.014).<br>85.9% of students agreed on the value of commercial films in teaching and 95.9% recommended the activity to other students.
Kim et al.<br><br>(2020)<br>[69]	Korea	Quasi-experimental study	92 first-year undergraduate medical students<br><br>C = 43<br>I = 49	**C**: 1- week of self-study<br><br>**I:** 1-hour of orientation on team-based learning, followed by 5 sessions of team-based tutorials which involved intra-group and inter-group discussion on quizzes related to the clinical pharmacological topics (e.g. antibiotics, pharmacodynamics)	Clinical pharmacology	**Objective**:<br>An individual knowledge test (10 MCQs)<br><br>A group knowledge test (10 MCQs)<br><br>A Group knowledge test (type not specified)<br><br>Comprehensive basic medical sciences exam (pharmacology section)<br><br>**Subjective**:<br>5-point Likert scale student evaluation of the intervention	 Before the intervention<br><br>During the intervention<br><br>Immediately after the intervention<br><br>4 weeks post intervention<br><br><br>Immediately after the intervention	 Level 2b<br><br>Level 2b<br><br>Level 2b<br><br>Level 2b<br><br><br>Level 1a, 1b	 The average score of both group knowledge tests were significantly higher than that of the pre-intervention test (p<0.001) which showed that the team-based learning achieved its intended learning outcomes. The team-based learning method had a statistically significant effect on low academic achievers from the intervention group (p=0.015). There was no difference in student performance between control and intervention groups overall (p=0.571).<br><br>In general, students' reactions were positive with a median score 4 out 5. However, students rated most negatively for pre-class preparation and knowledge tests for being challenging.
Kalikar et al.<br><br>(2020)<br>[72]	India	Cross-sectional study	192 Undergraduate medical students	1-hour lecture on the topic of pharmacovigilance and adverse drug reaction	Prescribing skills	**Objective**:<br>Tests on knowledge, awareness and attitudes towards pharmacovigilance and adverse drug reaction reporting in India<br><br>**Subjective**: NA	 Before and immediately after intervention	 Level 2a, 2b	 There was an overall improvement in knowledge, awareness and attitudes on pharmacovigilance and adverse drug reaction reporting (p<0.0001).
Nath et al.<br><br>(2021)<br>[73]	India	Cross-sectional interventional study	64 second-year undergraduate medical students	Students were divided into groups of 8 and each group was instructed to prepare two concept maps for a pharmacological topic. The concept maps were assessed, and the feedback was given to the students.<br><br>(Students were given 7 days to complete each concept mapping assignment.)	Clinical Pharmacology	**Objective**:<br>Concept mapping assignments<br><br>**Subjective**:<br>student evaluation on the intervention and open-ended feedback	 During the intervention<br><br>Immediately after the intervention	 Level 2b<br><br>Level 1a, 1b	 Overall, students performed better in the second concept mapping assignment.<br><br>Most students enjoyed the intervention and preferred concept mapping as a complementary learning tool. They opined that the intervention was helpful in organising their thoughts and bringing the gaps in their knowledge.

Remesh et al.<br><br>(2021)<br><br>(64)	India	Crossover study	64 second-year undergraduate medical students	**C**: Conventional teaching<br><br>**I**: An interactive teaching module on pharmacovigilance and adverse drug reaction monitoring, as per WHO Guide to Good Prescribing	Prescribing skills	**Objective**:<br>Knowledge tests (the type of questions was not specified)<br><br>**Subjective**:<br>5-point Likert scale student evaluation of the intervention	Immediately and 1 month after the intervention<br><br>Immediately after the intervention	Level 2b<br><br>Level 1a, 1b	Students from intervention group scored significantly higher than those from the control group (p<0.05) for both post-intervention knowledge tests. The retention of knowledge was also greater among students from the intervention group (p<0.05).<br>Overall, majority of the students were satisfied with the intervention. They agreed that the intervention was easy to follow and useful to help retain memory and build up confidence in prescription writing.

References

104. Kirsch V, Johannsen W, Thrien C, Herzig S, Matthes J. "Hopefully, I will never forget that again" - sensitizing medical students for drug safety by working on cases and simulating doctor-patient communication. GMS J Med Educ. 2019;36(2):Doc17.

105. Mozeika AM, Asri R, Theis JF, Suzuki CK. Pharmacology, pharmacotherapy, and pharmacopolicy through an evidence-based medicine: a novel approach for first-year medical students. MedEdPORTAL. 2020;16:10934.

106. Hasamnis AA, Arya A. Evaluation of teaching clinical pharmacology of antiarrhythmic drugs to first-year MBBS students through worksheet-based small-group discussion. J Pharm Bioallied Sci. 2017;9(4):282-3.

107. Gossell-Williams M, Paul T. Introducing medical students to pharmacovigilance through a basic research skills special study module. Int J Risk Saf Med. 2020;31(2):81-7.
